# Parental Correlates of Outdoor Play in Boys and Girls Aged 0 to 12—A Systematic Review

**DOI:** 10.3390/ijerph16020190

**Published:** 2019-01-11

**Authors:** Karolina Boxberger, Anne Kerstin Reimers

**Affiliations:** Institute of Human Movement Science and Health, Faculty of Behavioral and Social Sciences, Chemnitz University of Technology, 09111 Chemnitz, Germany; anne.reimers@hsw.tu-chemnitz.de

**Keywords:** physical activity, safety, restrictions, perceptions, neighborhood, outdoor play, children, parental correlates, gender, sex

## Abstract

Outdoor play is one major source of physical activity (PA) in children. In particular, parents act as gatekeepers, because they can enable their children’s outdoor play. This systematic review aims to provide an overview of parental correlates of outdoor play. A systematic literature research of six electronic databases (ERIC, PsycARTICLES, PsycINFO, PubMed/Medline, SCOPUS, and Web of Science Core Collection) was conducted with previously defined search terms, focusing on children 0–12 years old. In total, 1719 potentially publications were screened based on eligibility criteria. Included studies were scored for overall study quality. Findings were summarized using a semi-quantitative method. Twenty-one peer-reviewed publications which examined the relationship of parental correlates and outdoor play were included. Overall, five parental correlates were associated with children’s amount of outdoor play: mothers’ ethnicity, mothers’ employment status, parents’ education level, the importance parents assign to outdoor play, and perceived social cohesion in the neighborhood. Merely four studies reported sex/gender-stratified results. In summary, only parents’ encouragement/support provided evidence for girls’ amount of outdoor play. The findings are considered to be of public health relevance for developing intervention programs to increase outdoor play and for improving child’s health. More research, especially considering sex/gender of the child, is required.

## 1. Introduction

Regular physical activity (PA) provides several benefits for the health of children [1]. Therefore, many national and international guidelines have been developed to highlight the importance of PA in children. According to the Canadian 24-Hour Movement Guidelines for Children and Youth, children should perform at least 60 min of moderate-to-vigorous physical activity (MVPA) per day [2]. In accordance with the World Health Organization’s (WHO) guidelines, 5–12 year-olds should engage in at least 60 min of MVPA daily [3]. 

Outdoor play is one major source of PA in children [4]. Faulkner et al. [5] found that children who spend more than two hours outside per day reach a 27% higher level of MVPA compared to children who spend less time outdoors. According to Veitch et al. [6], outdoor play refers to any unorganized physical activity outdoors. Furthermore, outdoor play is freely chosen, spontaneous, self-directed, and includes activities the child enjoys [7]. In contrast to organized physical activities, like sports conducted in sports clubs, playing outdoors is often cheap and freely accessible [8]. 

Nevertheless, outdoor play has been declining over the last decades among children from western countries [9,10,11]. Hofferth and Sandberg [10] found that the daily time of outdoor play decreased by 25% from 1981 to 1997 in children aged 6–8 in the United States. More current data from Canada indicates a decline from 75% to 65% in outdoor playtime in children and youth from 2000 to 2010 [11]. Today, children spend less time outdoors than their parents during their childhood [9].

These findings are alarming, because in general children’s outdoor play is a “natural and critical part of a child’s healthy development” [9] and is connected with several health benefits: evidence revealed positive effects of outdoor play on cardiorespiratory fitness [12] and cardiovascular and metabolic health biomarkers [9], as well as overall health [13] and quality of life in children [14]. Furthermore, outdoor play is of high importance for their development, because it enables children to establish social contacts with peers [15]. During social interactions, children can learn skills such as social competence, risk management and creativity [16]—essential for their whole lives. Playing in natural environments like water or sand offers opportunities to gather (tactile) experiences [9] and develop a more positive relationship with the natural environment [17].

Children’s outdoor play should be promoted due to its benefits towards the healthy development of children and to stem its historical decline [18]. In this context, parents are considered to play an important role. According to Bandura’s Social Cognitive Theory [19], behavior is influenced by the behavior of others of importance. Parents are important and act as direct models for their children [20] and can influence their children’s PA and outdoor play. 

Furthermore, parents act as gatekeepers, because they can restrict or permit their children’s outdoor play [21]. Social support from parents is an important prerequisite for PA and outdoor play in children [22]. Parental motivation and encouragement are even “more important predictors of change” in time spent outside in 5–6-year-old children than the built environment [23]. In contrast, concerns about injuries during outdoor play are main reasons why parents forbid their children to play outdoors alone [24]. In particular, parents’ perception of safety was assumed as a determinant of children’s outdoor play [6]. According to a Canadian study of school-aged children, 82% of mothers mentioned concerns and fear about their children’s safety when playing outdoors without supervision [25]. Besides, on the basis of the Australian census data (2007–2013), the percentage of mothers who think that playing outdoors is dangerous has increased significantly, from 26% to 42% in children 2–3.5 years old [26]. 

Additionally, parenting styles are related to PA levels of children during outdoor play. Actually, Janssen [27] determined that parent’s supervision and hyper-parenting styles (“helicopter parents”; “little emperor parents”; “tiger moms”; parents who practice “concerted cultivation”) increased over time and were associated with lower PA during outdoor play time in 7–12 year-old children. Children whose parents had low levels of hyper-parenting styles had the highest outdoor play frequency scores [27]. Furthermore, if there is no supervising adult with them, children aged 10–11 accumulate higher amounts of PA during outdoor play [28].

Additionally, parents may act differently with their daughters and/or sons, respectively. With respect to the child’s sex/gender, boys’ parents are more likely to allow playing outdoors alone than girls’ parents [29]. Furthermore, boys are more active during all periods of the day—except evenings—and have a larger amount play time outside than their female counterparts [30]. 

In addition to biological theories on sexual differences, theoretical frameworks of gender socialization provide an explanation for gender differences in outdoor play. Gender refers to a cultural construction and equally a process of constructing ourselves [31]. Parents have the strongest influence on the development of their children’s gender roles [32]. From early childhood children learn what it means to be a boy or a girl. They develop a socio-culturally-determined gender identity [32] that leads to differences in health-related behaviors like PA [33] and outdoor play [30].

To date, health aspects of outdoor play have been largely examined: the systematic reviews of Gray et al. [34] and Brussoni et al. [35] found a relationship of outdoor play with physical fitness. The latest meta-analytical work of Truelove et al. [36] summarized and synthesized research findings about PA and sedentary time during outdoor play activities in young children (2–5 years). However, the relevance of parental correlates on outdoor play has not been summarized in a comprehensive and systematic way taking sex/gender differences into account. 

Therefore, the current systematic review aims to identify primary studies to synthesize the evidence on parental correlates of the amount of outdoor play in children aged 0–12 years old by considering sex/gender differences.

## 2. Materials and Methods 

### 2.1. Design

A systematic literature search was conducted to give an overview of the current state of research on parental correlates of outdoor play in children and to summarize primary studies. The systematic review follows the Preferred Reporting Items for Systematic Review and Meta-Analysis (PRISMA) [37]. 

### 2.2. Search Strategy

The systematic database query was performed on 23 April 2018. The search of relevant peer-reviewed journal articles was conducted in six electronic databases: in all fields in ERIC (limitation: peer-reviewed only), PsycARTICLES (limitations: peer-reviewed only, English or German language), PsycINFO (limitations: peer-reviewed only, English or German language), PubMed/Medline (limitation: academic journal articles), SCOPUS (limitations: English or German language), and in the topics in Web of Science Core Collection (limitations: English or German language). The database query was based on previously defined terms in different combinations. This terms were developed by using the Patient/Population, Intervention, Comparison and Outcome (PICO) strategy [38]. The following search terms and their variations were used: child, boy, girl, kid, preschooler, youth, toddler, infant, outdoor play, free play, unorganized play, active free play, unorganized physical activity, parent, mother, father, adult, maternal, and paternal. 

For documentation and for the screening of the literature retrieved in the data bases, all journal articles were exported to the literature management software EndNote (version X8, Analytics Clarivate, PA, USA). Additional articles were searched by considering the reference lists of the articles.

### 2.3. Eligibility Criteria

The a priori formulated eligibility criteria were as follows. Firstly, the study population had to consist of healthy children aged 0–12 or with the average age in this range. Secondly, the article had to present findings on the relationship of any parental factors with the amount of outdoor play in children (e.g., parents’ gender, parental encouragement, parents’ perception of the social or physical environment) displayed as minutes or hours per day or week. Thirdly, only studies with a quantitative design were included (e.g., cross-sectional, longitudinal; no qualitative studies). Fourthly, only peer-reviewed journal articles published in English and German language were included.

### 2.4. Parental Correlates

**Figure 1:**
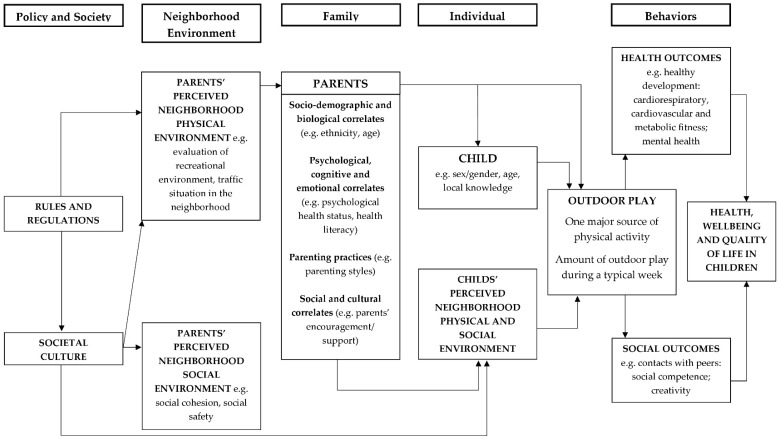
Socio-ecological model of different levels of influence on outdoor play in children (according to Sallis et al. [21]).

Based on the social ecological model of Sallis et al. [21], a new model was developed to depict parental attributes that might influence children’s outdoor play (see Figure 1). Referring to this model and previous systematic reviews [39,40] a classification of parental correlates in six different categories was conducted: (1) socio-demographic and biological correlates (e.g., age, ethnicity); (2) parents’ psychological, cognitive and emotional correlates (e.g., parents’ self-efficacy, depression); (3) parents’ social and cultural correlates (e.g., parental PA, parental encouragement); (4) parenting practices (e.g., presence of rules, hyper-parenting styles); (5) parents’ perceived physical environmental correlates (e.g., satisfaction with play facilities, perceived traffic situation); and (6) parents’ perceived social environmental correlates (e.g., social cohesion, social safety). 

### 2.5. Study Selection

The study selection process was done in three steps by two independent researchers: (1) title screening, (2) abstract screening, and (3) full-text screening. The studies were screened by considering the eligibility criteria. If all mentioned criteria have been met, the article was included in the systematic review. Any disagreement was solved by discussion of both reviewers and if necessary by also discussing with a third reviewer. Furthermore, the reference lists of all full-text-screened articles were used for citation chaining (forward and backward chaining) to ensure that all appropriate studies were captured. If there were slightest ambiguity regarding exclusion the study was taken over in the next step. Every step was documented in EndNote (X8).

### 2.6. Data Extraction

To give an overview of the characteristics of the included studies the following data were extracted from all articles: (1) Study characteristics (i.e., first author, publication year, country, study design, study sample description); (2) Definition and measurements of outdoor play and parental correlates; and (3) Main findings of parental correlates and outdoor play and sex/gender-related findings. Furthermore, all information necessary for the quality assessment was extracted. An overview of all extracted data is presented in Supplementary material Table S1 (overall study description and main multivariate results) and Supplementary material Table S2 (overall study description and main sex/gender-stratified results).

### 2.7. Quality Assessment

Two independent researchers conducted the methodological quality assessment of the studies. As several included publications were cross-sectional studies, a modified version of the Appraisal tool for Cross-Sectional Studies (AXIS) developed in a Delphi process [41] was used for scoring the quality of the studies. 

Originally, the tool has five sections: introduction, methods, results, discussion and other. In this review the items 1–18 were used to assess the overall quality of the studies (criteria about sample size, target population, selection process, statistically methods or data description). Items 19 and 20 were removed, because they were not suitable for rating the methodological quality of the primary studies in the present review. To score the response rates item 13 was fulfilled if the response was ≥60% [42]. 

In line with previous reviews, each criterion was rated as yes = 1 if the criterion was fully fulfilled, partial = 0.5 if the criterion was partially fulfilled, or as no/unclear/not applicable = 0 [42,43]. A composite score was calculated as the percentage of quality criteria (*N* = 18) that were fulfilled. Study quality with a score of ≥66.7% was classified as “high”, between 50 and 66.6% as “fair”, and below 50% as “low” [44].

### 2.8. Analysis and Synthesis of Results

A meta-analysis was excluded due to heterogeneity of the included studies. A semi-quantitative synthesis of the results was conducted. Associations of parental correlates and outdoor play were summarized and synthesized for overall samples. Furthermore, to take sex/gender-related similarities and differences into account, associations of parental correlates and outdoor play were analyzed separately for boys and girls. In line with previous systematic reviews only adjusted associations between parental correlates and outdoor play in children were considered in the present review [45,46]. 

Due to the fact that studies reported relevant findings for boys and girls separately, considered different age groups, samples were independently included in the analyses as done in a previous review [47]. In one study [48], correlates of children’s and parents’ reports of outdoor play were analyzed separately. The findings of these self- and proxy-reported outdoor play correlates were separately included in this review, because the correlation of children’s and parents’ reported outdoor play has been shown to be low [48]. 

The scoring system to measure the strength of evidence was based on a modification of previously published scoring systems [40,47,49,50]. Positive (+) or negative (−) associations were assumed if 60–100% of the samples analyzed provided significant associations in the same direction. If 60–100% of the included studies were of high quality, the findings were considered as strong evidence. If only 34–59% of the samples analyzed reported significant associations, the findings were rated as inconsistent in the expected direction. No association was classified if 0–33% of the samples showed a significant association between a parental correlate and outdoor play. Additionally, in line with previous studies, if fewer than three samples were available to describe an association the evidence was rated as limited [42,47].

## 3. Results

### 3.1. Flow Chart

**Figure 2:**
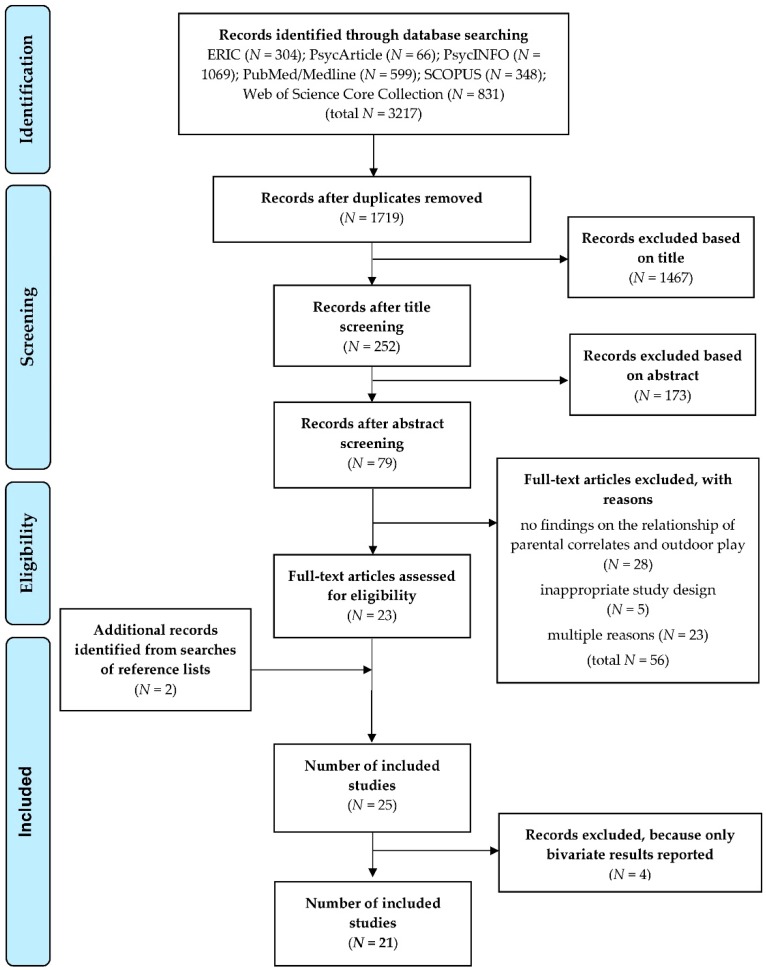
Preferred reporting items for systematic reviews and meta-analyses (PRISMA) flow diagram [37] presenting the results of the research, screening and selection processes of the present review.

A total of 3217 publications were identified through database searching. Duplicates (*n* = 1498) were removed from the library, resulting in 1719 research articles included in the screening process. Overall, 23 publications fulfilled the eligibility criteria. Additionally, two studies were identified from searches of reference lists, resulting in 25 articles. A posteriori, four studies [51,52,53,54] were excluded because they reported bivariate results only. Thus, finally 21 studies were included in this review, reporting data on 30 independent samples in total. The process of the study selection is depicted in Figure 2. 

### 3.2. Study Characteristics of Included Studies

Of the 21 studies included in this review in total, 17 studies had a cross-sectional and four a longitudinal design. Seven studies were conducted in Europe and four in Australia. However, the vast majority of the studies (*N* = 11) were conducted in America. 

With the exception of four publications, most articles were published within the last eight years. More than a half of the included studies had a sample size markedly lower than 1000 children. Overall, the sample sizes ranged from 421 [23] to 8950 [55]. All studies targeted both boys and girls, but only four articles reported results separated by sex/gender [23,48,56,57]. In merely six of the 21 studies were there more than 50% female participants. 

A predominant proportion of studies relied on parental reports based on questionnaires to assess outdoor play in children. Only one study considered children’s as well as parental reported outdoor play [48]. More descriptive information of the included studies is presented in Table 1.

### 3.3. Results of Study Quality Assessment

The study quality was rated high in 11 [27,48,56,57,58,63,64,65,66,67,69] and fair in 10 [5,23,26,55,59,60,61,62,68,70] studies. The more detailed quality assessment for each study is presented in the Supplementary material Table S3. The two independent reviewers agreed in 85% (Cohen’s kappa κ = 0.851). The results of the quality assessment in relation to each criterion are presented in Table 2. 

The following criteria were met by most of the primary studies included in this review: All studies stated a clearly defined target population. Nearly all publications appropriately described aims/objectives and provided clear and consistent results to their aims. Additionally, almost all studies used appropriate correlates and outcomes. 

In contrast, the following criteria were rarely met: A predominantly part of studies used measurements with a low reliability or did not mentioned the reliability of used measurements. Only Nicksic et al. [48] used reliable measurements in their study. Moreover, in merely five publications were the socio-demographic characteristics of non-responders analyzed appropriately by using statistical methods [48,57,63,66,69]. However, no study undertook measures to address specific groups of non-responders. Simply eight studies acquired a response rate of at least 60% [5,48,56,58,59,63,66,67]. Additionally, in three publications results were not reported by the authors or they were shortly mentioned elsewhere in the results section but not presented in tables [26,62,70].

### 3.4. Parental Correlates of Outdoor Play in Children

Table 3 presents all identified parental correlates divided into categories and sub-categories and their level of evidence. Several studies provided specific results for maternal and paternal correlates and are presented separately (e.g., mother’s age, father’s age). In total, the results of this systematic review show that different parental correlates of the socio-demographic, psychological, cognitive and emotional, and neighborhood social environmental levels are associated with outdoor play in children aged 0–12. 

Overall, 15 socio-demographic and biological correlates were identified in 15 different studies by considering 55 independent samples. Evidence was found for mothers’ ethnicity and mothers’ employment status as well as parents’ education level in the expected direction. 

In total, nine parents’ psychological, cognitive and emotional factors were analyzed in five studies and in overall 22 independent samples. Only parents’ perception of importance of outdoor play was significantly associated with children’s amount of outdoor play. 

Relationships between parenting practices and outdoor play were analyzed in six different studies. Totally, four variables of parenting practices were examined by considering 22 independent samples. No significant associations in the expected directions for parenting practices with outdoor play were found. The same is true for the six parental social and cultural correlates identified in nine different studies and 30 independent samples overall. 

Respectively, 13 different correlates concerning parents’ perceived neighborhood physical environment were identified in nine studies by considering 79 independent samples. This was the most frequently examined category of parental correlates. Nearly no significant associations were found in this category—except for the availability of suitable play facilities in the neighborhood. 

In 11 studies and 40 independent samples, two different correlates of parents’ perceived neighborhood social environment were identified. Parents’ perceptions of social safety in the neighborhood were not significantly associated with the amount of outdoor play in children. However, parents’ perceptions of social cohesion in the neighborhood were related to children’s outdoor play in the expected direction.

### 3.5. Sex/Gender-Related Results of Parental Correlates and Outdoor Play

With respect to sex/gender-related findings, 29 independent samples in four different studies were analyzed separately for boys and girls and examined for ten different potential correlates. In terms of the relationship between parents’ encouragement/support and outdoor play, a consistent relationship was found in girls only [23,48]. 

However, the analyses concerning parental supervision, outdoor social opportunities [23], and parents perceptions of diversity of routes [56] provided inconsistent results. These correlates were significantly associated with outdoor play in boys and girls in different specific age groups. All other variables provided limited evidence, as these correlates were studied less frequently.

## 4. Discussion

The aim of this systematic review was to identify and synthesize parental correlates of outdoor play in children 0–12 years old by taking sex/gender-related aspects into account. Outdoor play is related to several health benefits in children [9,12,14]. Actually, researches highlight the alarming decline of outdoor play in children from western countries like America and Canada [9,10,11]. Thus, the results of this systematic work are considered to be of public health relevance, because these could inform policy and public health societies to develop intervention programs for increasing outdoor play in children. Overall, the results of this systematic review show, that the amount of children’s outdoor play is associated with different parental correlates of the following categories: parents’ socio-demographic and biological correlates, psychological, cognitive and emotional correlates, and parents’ perceptions of neighborhood social environment. 

For instance, the findings of this review demonstrated that children of mothers of an ethnic minority played less time outdoors than children of mothers from the ethic majority. Studies found that women of a minority ethnicity are less physically active than women of majority ethnicity [73], thereby infrequently acting as a physically active role model for their children by supporting PA and outdoor play. Additionally, ethnic minority groups more likely live in deprived residential areas [74]. Researchers confirmed that mothers living in a poor neighborhood reported more fears of children’s outdoor play than those from non-deprived residential areas [75]. For these reasons, mothers of an ethnic minority possibly restrict their children’s outdoor play more than mothers from the ethnic majority. 

Furthermore, if mothers had a full-time job their children’s amount of outdoor play was lower than of children with unemployed mothers. Overall, female biography changed during the last century: education and employment became more common among women [76]. Therefore, women are trying to create a balance between their roles as mothers and workers [77]. Simultaneously, daycare attendance of non-familial institutions has increased [78]. For instance, 40% of the children aged 3–4 of employed mothers in the United States were in non-familial childcare over 35 h per week [79]. Possibly, mothers expect that daycare centers facilitate opportunities to be physically active and to play outdoors for their children [61]. Mothers encouragement of their children in playing outdoors at home may have decreased due to these reasons [61]. 

Additionally, parents’ high education level is negatively related to outdoor play in children. Children of highly educated parents refer a higher logistical and financial support than children of parents with less education [80]. Parents with a high education level more likely engage their children to participate in organized physical activities and family-based activities, like swimming or dog-walking [80], resulting in lower amounts of outdoor play. In contrast, children of less-educated parents reported a higher amount of unstructured activities—like outdoor play—due to the monetary costs [80]. 

Overall, the study of Watchman and Spencer-Cavaliere [81] highlighted that organized physical activities were prioritized by parents because they were perceived as more important for children’s development than playing outdoors. However, this review showed that the importance parents placed on outdoor play was positively related to their children’s amount of outdoor play. The frequency and amount of playing in parks were also higher if parents felt that playing in a park playground was important for their children’s PA [82]. This indicates that the knowledge of benefits of outdoor play as an important source of children’s PA may influence parents to encourage it. 

Moreover, mothers who live in poor neighborhoods have fewer social relationships [83] and as a consequence benefit to a lesser extent from social support from the people living in their neighborhood [84]. In contrast, neighborhoods with higher social cohesion are characterized by strong social bonds and low social conflicts between the inhabitants [85]: social relationships that exist among parents [86] may facilitate outdoor play in the neighborhood, as parents know and trust their neighbors and are communicating with each other [87]. This could explain the result of this review demonstrating that parents’ perception of a high social cohesion in the neighborhood is positively related to a child’s amount of outdoor play. 

A variety of previous systematic reviews underlines the importance of parental encouragement and social support as a key factor for children’s PA [39,40]. However, the present review revealed that this encouragement/support is only related to the amount of outdoor play of girls but not boys. 

Parents are more likely to allow boys to play outdoors alone than girls [29]. From early childhood onwards, parents seem to be more protective of their daughters than sons [88]. Additionally, fear of strangers or traffic danger is greater in parents of girls than boys [29]. It is the same regarding concerns about molestation and assault [89]. Therefore, parents are more likely to restrict unsupervised outdoor play in girls. Additionally, parents communicate differently with boys and girls about injury-risking behavior on playgrounds [88]. For example, parents requested boys to reach the pole without parental assistance more often than girls [88]. The authors concluded that parents have greater expectations for boys to manage injury-risking situations than for girls [88]. Consequently, during outdoor play girls are less likely than boys to exhibit behavior perceived as connected with injuries [90]. Thus, girls prefer indoor activities [88] and spend more time playing indoors in more static types of play, while boys spend more time on outdoor activities [91]. 

These aspects indicate that girls are more dependent on parents’ encouragement/support than boys. However, girls receive less social support from significant others—like parents or peers—than boys [92]. Thus, intervention programs should focus on parental encouragement/support to increase outdoor play and PA in girls.

### 4.1. Methodological Limitations of Studies Reviewed

Due to heterogeneity of the methodological approaches of the primary studies the results of parental correlates and outdoor play in children may be influenced by several aspects. To begin with, different populations were interviewed to assess outdoor play. The majority of studies used parental report questionnaires. Two studies [57,65] measured children’s self-reported outdoor play via questionnaires, while only one study measured outdoor play by using both self and proxy reports [48]. Moreover, outdoor play was measured by focusing on different aspects. Some researchers assessed outdoor play duration in questionnaires for different intensities (e.g., quiet, moderate and vigorous outdoor play) and summed up the durations [59] while others merely inquired over duration of children’s overall outdoor play [23,52,56,58,64]. Furthermore, in other studies outdoor play was captured by asking parents how long their child played in different locations (e.g., the yard in someone else’s home, the street or cul-de-sac the home was on, parks, and playgrounds outside of school hours) [27,62]. 

Due to methodological inconsistencies, further research is necessary to develop a clear definition of outdoor play that guides the development of internationally accepted and distributed instruments for the measurement of outdoor play. The usage of standardized methods and instruments could enhance comparison of results across studies. Bates and Stone [43] postulated that a combination of objective and subjective methods would be the optimum.

### 4.2. Strengths and Limitations of the Present Review

There are numerous strengths and limitations concerning the present systematic review. This is the first systematic and comprehensive summary of studies on parental correlates of outdoor play in children. Several databases were used for the literature search to ensure the identification of all existing journal articles about the research question on hand and reliability of the screening process was verified by engaging two independent reviewers. Additionally, a standardized risk of bias tool [41] developed especially for cross-sectional studies was used to score the quality of the included studies and to evaluate the risk of bias. This tool was modified to score overall, reporting and methodological quality of study. 

Nevertheless, a limitation of the review was that only studies in German or English language could be included. Articles published in other languages were excluded, leading possibly to a language bias. Furthermore, the bias against publishing negative findings may have affected the results of the present review. Overall, all presented results were reported with different statistical parameters and only assessed by significance and direction. However, the results of this review should be interpreted carefully, because several parental correlates were reported only by a low number of studies. For instance, the parental correlate “the importance of outdoor play” was investigated in different age-groups of one study. These limitations and the heterogeneity of parental correlates limit the generalizability of the results of this review. Additionally, although 1719 references identified by database queries were reviewed by two independent researchers, it is possible that articles were overlooked in the screening process.

## 5. Conclusions

This systematic review provides an overview of parental correlates of outdoor play in children 0–12 years old. Based on socio-ecological models, this research identified socio-demographic, psychological, cognitive and emotional and neighborhood social environmental parental correlates of outdoor play in children. Overall, this review pointed out that the mother’s employment status and ethnicity as well as parents’ education level, perceptions of social cohesion in the neighborhood, and importance they assign to outdoor play are associated with outdoor play in children. Thus, these aspects should be considered for promoting outdoor play. As children of ethnic minorities and children of employed mothers were identified as enjoying a lower amount of outdoor play, these populations in particular should be the target of interventions. 

Additionally, the synthesis of existing studies considering sex/gender-stratified results revealed that parental encouragement/support was important for outdoor play in girls only. Thus, outdoor play in girls could specifically be promoted by increasing social support and encouragement from the parents. Based on a large body of evidence on lower levels of PA among girls compared to boys, with a growing gap in the transition from childhood to adolescence [40], more sex/gender-specific research is required to develop successful gender-sensitive intervention programs.

## Figures and Tables

**Table 1:** Characteristics of included studies (*N* = 21).

Characteristics	Study Source	*N* (%)
**Study design**		
Cross-sectional	[5,26,27,48,55,56,57,58,59,60,61,62,63,64,65,66,67]	17 (81)
Longitudinal	[23,68,69,70]	4 (19)
**Country of studies ^a^**		
Australia	[23,26,67,70]	4 (19)
Canada	[5,27,61]	3 (14)
Netherlands	[56,58,66,68,69]	5 (24)
Switzerland	[59]	1 (5)
United Kingdom	[57]	1 (5)
United States	[27,48,55,60,62,63,64,65]	8 (38)
**Publication year**		
2000–2010	[23,56,59,60]	4 (19)
2011–2018	[5,26,27,48,55,57,58,61,62,63,64,65,66,67,68,69,70]	17 (81)
**Sample size**		
>1000	[55,56,58,60,61,63,64,66,68,69]	10 (48)
<1000	[5,23,26,27,48,57,59,62,65,67,70]	11 (52)
**Population’s (average) age**		
0–6	[26,55,60,61,63,64,66,67,68,69,70]	11 (52)
7–12	[5,27,56,57,58,62,65]	7 (34)
not reported/countable	[23,48,59]	3 (14)
**Sex/gender proportion**		
female > 50%	[5,48,57,62,65,67]	6 (29)
female < 50%	[27,55,56,58,59,60,61,63,64,66,68,69]	12 (57)
not reported/countable or variable	[23,26,70]	3 (14)
**Outdoor play measured in**		
Duration	[5,23,26,56,57,58,59,60,61,63,64,67,70]	13 (62)
Frequency	[27,48,55,62,65]	5 (24)
Frequency and duration	[66,68,69]	3 (14)
**Outdoor play measured for**		
Weekdays	[61,63,64]	3 (14)
Week and weekend days	[5,23,26,27,48,55,56,57,58,59,60,62,66,67,68,69,70]	17 (81)
Not specified (past five days measured)	[65]	1 (5)
**Outdoor play report type**		
Child reported	[57,65]	2 (10)
Parent reported	[5,23,26,27,55,56,58,59,60,61,62,63,64,66,67,68,69,70]	18 (86)
Child and parent reported	[48]	1 (5)

*Note: N* = number of studies; a = The study of Janssen [27] was based on an international recruitment from the FluidSurvey^TM^ panel and was considered for both countries: United States and Canada.

**Table 2:** Criteria for quality assessment and number (%) of studies scoring points for each criterion.

Criterion	Description	Number of Studies Fulfilling the Criteria *N* (%)	
		Fulfilled	Partial Fulfilled
Introduction			
1. Objectives	Were the aims/objectives of the study clearly described?	18 (86)	3 (14)
Methods			
2. Study design	Was the study design appropriate for the stated aim(s)?	10 (48)	11 (52)
3. Sample size justification	Was the sample size justified?	3 (14)	2 (10)
4. Definition of target population	Was the target/reference population clearly defined?	21 (100)	0 (0)
5. Sampling frame	Was the sample frame taken from an appropriate population base so that it closely represented the target/reference population under examination?	7 (33)	14 (67)
6. Sample selection process	Was the selection process likely to select subjects/participants that were representative of the target/reference population under examination?	4 (19)	16 (76)
7. Address of non-responders	Were measures undertaken to address non-responders?	0 (0)	0 (0)
8. Appropriateness of aim(s) and outcome	Were the correlates and outcome variables measured appropriate to the aims of the study?	18 (86)	3 (14)
9. Appropriateness of measurements	Were the correlates and outdoor play measured correctly using measurements that had been trialled, piloted or published previously? (intra-class correlation coefficient: ICC ≥ 0.75 (good reliability = criterion fulfilled) [71]/Cronbach’s α ≥ 0.8 (good reliability = criterion fulfilled) [72] ^a,b^	1 (5)	8 (38)
10. Clearness of statistical significance	It is clear what was used to determined statistical significance?	13 (62)	7 (33)
11. Sufficiently description of methods	Were the methods sufficiently described to enable them to be repeated?	13 (62)	8 (38)
Results			
12. Adequately description of basic data	Were the basic data adequately described?	17 (81)	4 (19)
13. Response rate	Was the response rate 60% or more? ^c^	8 (38)	0 (0)
14. Description of non-responders	If appropriate, was information about non-responders described?	5 (24)	6 (29)
15. Consistent results	Were the results internally consistent?	19 (90)	2 (10)
16. Completeness of results	Were the results presented for all the analyses described in the methods?	7 (33)	11 (52)
Discussion			
17. Justified results	Were the discussion and conclusions justified by the results?	15 (71)	6 (29)
18. Limitations	Were the limitations of the study discussed?	19 (90)	2 (10)

*Note:*^a^ = ICC and Cronbach’s α values based on Veitch et al. [71]; George and Mallery [72]; ^b^ = If reliability of different correlates ranged from poor to high in the same study, a value of 0.5 points was assigned. ^c^ = in accordance to Schoeppe et al. [42].

**Table 3:** Parental correlates of outdoor play in children.

Parental Correlates of Outdoor Play	Association with Outdoor Play			Strength of Evidence	
	+	0	−	*n/N* (%)	Association
**Socio-demographic and biological correlates**					
**Individual characteristics**					
*Mother*					
Age		[63] ^a^	[67]	1/2 (50)	N/A
Body mass index		[63] ^a^		1/1 (100)	N/A
Ethnicity (ethnic minority)		[61] ^a,d^	[61] ^a,d^; [55]; [66]; [70]; [26] ^a,d^; [26] ^a,d^; [26] ^b,d^; [26] ^b,d^	8/9 (100)	-
*Father*					
Age		[68] ^d^; [68] ^d^		2/2 (100)	N/A
*Parents*					
Ethnicity (ethnic majority)		[68] ^d^; [68] ^d,e^		2/2 (100)	N/A
Body mass index	[68] ^d^; [68] ^d^			2/2 (100)	N/A
**Family status**					
Marital status (married)			[67]	1/1 (100)	N/A
Family structure (single parent household)	[63] ^a^	[62]		1/2 (50)	N/A
**Socio-economic status**					
*Mother*					
Education level (high)		[63] ^a^; [61] ^a,d^; [61] ^a,d^; [59];	[66]; [26] ^a,d^	4/6 (67)	0
Employment status		[66]	[63] ^a^; [55]	2/3 (67)	−
*Father*					
Education level		[66]		1/1 (100)	N/A
Employment status		[66]		1/1 (100)	N/A
*Parents*					
Education level (high)		[68] ^d^; [62]; [58] ^d^; [57] ^b,c^	[55]; [68] ^d^; [58] ^d^; [58] ^d^; [57] ^a^; [56] ^d^; [56] ^d^; [56] ^d^;	8/12 (67)	−−
Employment status		[5] ^a,c^	[5] ^b^	1/2 (50)	N/A
Household income (low)	[66]	[63] ^a^; [61] ^a,d^; [61] ^a,d^; [62]		4/5 (80)	0
**Parents’ psychological, cognitive and emotional correlates**					
**Psychological health status**					
*Mother*					
Depression		[63]		1/1 (100)	N/A
**Health literacy**					
*Mother*					
Knowledge about child development		[70] ^e^		1/1 (100)	N/A
Knowledge about playing with child	[70] ^b,e^	[70] ^a,e^		1/2 (50)	N/A
*Parents*					
Concerns of child’s obesity		[70] ^e^	[69] ^e^	1/2 (50)	N/A
**Attitude towards child’s recreation**					
*Parents*					
Importance of outdoor play	[56] ^d^; [56] ^d^; [56] ^d^			3/3 (100)	++
Outdoor play as habit	[68] ^d^; [68] ^d^			2/2 (100)	N/A
Attitude for improving outdoor play		[70] ^e^; [68] ^d^; [68] ^d^; [68] ^d,e^; [69] ^e^	[68] ^d^; [68] ^d^; [68] ^d,e^	5/8 (63)	0
Perceptions of difficulty for improving child engagement in outdoor play			[68] ^d^; [68] ^d^	2/2 (100)	N/A
Parental attitude towards child’s PA	[69] ^e^			1/1 (100)	N/A
**Parenting practices**					
**Psychological aspects of parenting**					
Parental hostility	[67] ^c^			1/1 (100)	N/A
**Parenting styles**					
Presence of rules and restrictions	[70] ^d^; [68] ^d^; [68] ^d^; [68] ^d,e^	[70] ^d^; [56] ^d^; [56] ^d^; [56] ^d^	[69] ^e^	4/9 (75)	?
Hyper-parenting	[68] ^d^; [27] ^c^	[68] ^d^; [69] ^e^		2/4 (50)	?
Parental supervision		[68] ^d^; [68] ^d^; [62]; [69] ^e^; [23] M ^c,d^; [23] F ^c,d^;	[23] M ^c,d^; [23] F ^c,d^	6/8 (75)	0
**Parents’ social and cultural correlates**					
**Encouragement and social support**					
*Parents*					
Encouragement/support (high)	[62]; [65]; [48]; [23] F ^d^; [23] F ^d^; [48] F	[68] ^d^; [23] M ^d^; [23] M ^d^; [48] M	[68] ^d^	6/11 (55)	+?
**Modeling**					
*Mother*					
PA	[55]; [70] ^b,e^	[70] ^a,e^; [26] ^a,d^; [26] ^b,d^; [26] ^a,d^; [26] ^b,d^		5/7 (71)	0
*Parents*					
PA		[68] ^d^; [68] ^d^; [62]		3/3 (100)	0
Partner’s PA	[68] ^d^	[68] ^d^		1/2 (50)	N/A
**Activities with child**					
*Parents*					
Involvement in PA with child	[62]			1/1 (100)	N/A
Outdoor social opportunities	[64]; [23] M ^d^	[23] M ^d^; [23] F ^d^; [23] F ^d^		3/5 (60)	0
**Parents’ perceived neighborhood physical environment**					
**Evaluation of recreational environment**					
Attractiveness of physical recreational environment	[26] ^a,d^; [26] ^b,d^; [26] ^b,d^	[26] ^a,d^; [68] ^d^; [68] ^d^; [68] ^d^; [68] ^d^; [69] ^e^; [65]		7/10 (70)	0
Satisfaction with physical recreational environment		[56] ^d^; [56] ^d^; [56] ^d^; [56] ^d^; [56] ^d^; [56] ^d^		6/6 (100)	00
Perceptions of cleanliness of the neighborhood	[56] F ^c,d^	[56] ^d^; [56] ^d^; [56] ^d^; [56] ^d^; [56] ^d^; [56] ^d^; [56] M ^d^		7/8 (88)	00
**Availability of recreational environment**					
Availability of suitable play facilities in neighborhood	[59]; [59]; [59]; [26] ^a,d^; [26] ^b,d^; [69] ^e^	[26] ^a,d^; [26] ^b,d^; [56] ^c,d^; [56] ^c,d^; [56] ^c,d^		6/11 (55)	+?
Perceptions of the degree of natural environment	[56] M ^d^	[56] ^d^; [56] ^d^; [56] ^d^; [56] ^d^; [56] ^d^; [56] F ^d^		6/7 (86)	00
Perceptions of the degree of high-rise buildings		[56] ^d^; [56] ^d^; [56] ^d^		3/3 (100)	00
Perceptions of the degree of unoccupied houses	[56] M ^d^	[56] ^d^; [56] ^d^; [56] F ^d^		3/4 (75)	00
**Traffic situation in the neighborhood**					
Perceptions of heavy traffic situation	[62] ^c,f^; [62] ^f^	[63] ^a,c^; [26] ^a,d^; [26] ^a,d^; [26] ^b,d^; [26] ^b,d^; [57] ^a^; [57] ^b^; [56] ^d^; [56] ^d^; [56] ^d^; [5] ^a^; [5] ^b^; [5] ^b^; [69] ^e^; [57] F	[59]; [5] ^a^; [57] M	15/20 (75)	0
Perceptions of the quality of footpaths and bike lanes		[56] ^d^; [56] ^d^; [56] ^d^; [69] ^e^		4/4 (100)	00
Perceptions of the diversity of routes	[56] F ^d^; [56] M ^d^	[56] ^d^; [56] M ^d^; [56] F ^d^		3/5 (60)	00
Attractiveness of roads		[5] ^a^; [5] ^b^		2/2 (100)	N/A
**Parents’ perceived neighborhood social environment**					
**Social relationships in the neighborhood**					
Social cohesion	[56] ^d^; [56] ^d^; [5] ^a^; [5] ^b^; [69] ^e^; [56] F ^d^	[56] ^d^; [56] ^d^; [56] ^d^; [56] M ^d^		6/10 (60)	++
**Safety perceptions in the neighborhood**					
Social safety	[67]; [26] ^a,d^; [26] ^b,d^; [26] ^b,d^; [62] ^cdg^; [62] ^g^; [57] ^a,c^; [56] ^d^; [48] M	[26] ^a,d^; [26] ^a,d^; [26] ^a,d^; [26] ^b,d^; [26] ^b,d^; [68] ^d^; [68] ^d^; [68] ^d^; [68] ^d^; [57] ^b,c^; [56] ^d^; [56] ^d^; [5] ^a^; [5] ^b^; [48]; [60] ^a^; [60] ^b^; [48] F	[59] ^c^; [5] ^a,c^; [5] ^b,c^	18/30 (60)	0

*Note:* M = male; F = female; *n/N* = sample size; % = percent of samples showed an association in the expected direction; (+) = positive association, 60–100% of samples showed a (significant) association in the expected direction; (−) = negative association, 60–100% of samples showed a (significant) association in the expected direction; (+?), (−?), or (0?) = 34–59% of samples showed a (significant) association in the expected direction; (0) = no association, 0–33% of samples showed a (significant) association in the expected direction; (++), (−−), or (00) = 60–100% of high quality study samples showed a (significant) association in the expected direction; (N/A) = <3 samples; ^a^ = results considered weekdays and weekend days separately; ^b^ = results considered weekend days; ^c^ = reversed items; ^d^ = results for separately analyzed age groups; ^e^ = longitudinal results; ^f^ = presented associations include two traffic-related items (i.e., unsafe road factor and traffic calming factor); ^g^ = presented associations include two social safety-related items (i.e. perceptions of crime risk and outdoor play is safe for children). PA: physical activity.

## Supplementary Materials

The following are available online at http://www.mdpi.com/1660-4601/16/2/190/s1, Table S1: Extracted data of studies included in systematic review (multivariate findings), Table S2: Extracted data of studies included in systematic review (gender-related findings), Table S3: Quality assessment for all included studies.

## References

[1] Janssen I., Leblanc A.G. (2010). Systematic review of the health benefits of physical activity and fitness in school-aged children and youth. Int. J. Behav. Nutr. Phys. Act..

[2] Tremblay M.S., Carson V., Chaput J., Connor Gorber S., Dinh T., Duggan M., Faulkner G., Gray C.E., Gruber R., Janson K. (2016). Canadian 24-Hour Movement Guidelines for Children and Youth: An Integration of Physical Activity, Sedentary Behavior, and Sleep. Appl. Physiol. Nutr. Metab..

[3] World Health Organization Physical Activity—How Much of Physical Activity Is Recommended?. http://www.who.int/en/news-room/fact-sheets/detail/physical-activity.

[4] Ansari A., Pettit K., Gershoff E. (2015). Combating obesity in head start: Outdoor play and change in children’s body mass index. J. Dev. Behav. Pediatr..

[5] Faulkner G., Mitra R., Buliung R., Stone M. (2015). Children’s outdoor play time, physical activity, and parental perceptions of the neigbourhood environment. Int. J. Play.

[6] Veitch J., Bagley S., Ball K., Salmon J. (2006). Where do children usually play? A qualitative study of parents’ perceptions of influences on children’s active free-play. Health Place.

[7] Bergen D. (2009). Play as the learning medium for future scientists, mathematicians, and engineers. Am. J. Play.

[8] Farley T.A., Meriwether R.A., Baker E.T., Watkins L.T., Johnson C.C., Webber L.S. (2007). Safe play spaces to promote physical activity in inner-city children: Results from a pilot study of an environmental intervention. Am. J. Public Health.

[9] Clements R. (2004). An investigation of the status of outdoor play. CIEC.

[10] Hofferth S.L., Sandberg J.F. (2001). Changes in American children’s time, 1981–1997. Adv. Life Course Res..

[11] Canadian Fitness and Lifestyle Research Institute Bulletin 04: Children’s Active Pursuits during the after School Period. http://www.cflri.ca/sites/default/files/node/922/files/PAM%202010%20Bulletin%204%20-%20Active%20Pursuits%20EN.pdf.

[12] Schaefer L., Plotnikoff R.C., Majumdar S.R., Mollard R., Woo M., Sadman R., Rinaldi R.L., Boulé N., Torrance B., Ball G.D.C. (2014). Outdoor time is associated with physical activity, sedentary time, and cardiorespiratory fitness in youth. J. Pediatr..

[13] Brussoni M., Olsen L.L., Pike I., Sleet D.A. (2012). Risky play and children’s safety: Balancing priorities for optimal child development. Int. J. Environ. Res. Public Health.

[14] Bento G., Dias G. (2017). The importance of outdoor play for young children’s healthy development. Porto Biomed. J..

[15] Dowdell K., Gray T., Malone K. (2011). Nature and its influence on children’s outdoor play. AJOE.

[16] Back J., Heeffer C., Paget S., Rau A., Sallnäs Pysander E.L., Waern A. Designing for Children’s Outdoor Play. Proceedings of the 2016 ACM Conference on Designing Interactive Systems.

[17] Bixler R.D., Floyd M.F., Hammitt W.E. (2002). Environmental socialization: Quantitative tests of the childhood play hypothesis. Environ. Behav..

[18] Gray P. (2011). The decline of play and the rise of psychopathology in children and adolescents. Am. J. Play.

[19] Bandura A. (2004). Health promotion by social cognitive means. Health Educ. Behav..

[20] Griffith J.R., Clasey J.L., King J.T., Gantz S., Kryscio R.J., Bada H.S. (2007). Role of parents in determining children’s physical activity. World J. Pediatr..

[21] Sallis J.F., Cervero R.B., Ascher W., Henderson K.A., Kraft M.K., Kerr J. (2006). An ecological approach to creating active living communities. Annu. Rev. Public Health.

[22] Beets M.W., Vogel R., Chapman S., Pitetti K.H., Cardinal B.J. (2007). Parent’s social support for children’s outdoor physical activity: Do weekdays and weekends matter?. Sex Roles.

[23] Cleland V., Timperio A., Salmon J., Hume C., Baur L.A., Crawford D. (2010). Predictors of time spent outdoors among children: 5-year longitudinal findings. J. Epidemiol. Community Health.

[24] Evans J. (2000). Where do the children play?. C. Aust..

[25] Barnes J.D., Colley R.C., Tremblay M.S. (2012). Results from the Active Healthy Kids Canada 2011 report card on physical activity for children and youth. Appl. Physiol. Nutr. Metab..

[26] Xu H., Wen L.M., Hardy L.L., Rissel C. (2017). Mothers’ perceived neighbourhood environment and outdoor play of 2- to 3.5-year-old children: Findings from the healthy beginnings trial. Int. J. Environ. Res. Public Health.

[27] Janssen I. (2015). Hyper-parenting is negatively associated with physical activity among 7–12 year olds. Prev. Med..

[28] Page A.S., Cooper A.R., Griew P., Davis L., Hillsdon M. (2009). Independent mobility in relation to weekday and weekend physical activity in children aged 10-11 years: The PEACH Project. Int. J. Behav. Nutr. Phys. Act..

[29] Soori H., Bhopal R.S. (2002). Parental permission for children’s independent outdoor activities. Implications for injury prevention. Eur. J. Public Health.

[30] Carver A., Timperio A., Hesketh K., Crawford D. (2010). Are children and adolescents less active if parents restrict their physical activity and active transport due to perceived risk?. Soc. Sci. Med..

[31] Butler J. (1986). Sex and Gender in Simone de Beauvoir’s Second Sex. Yale Fr. Stud..

[32] Witt S.D. (1997). Parental influence on children’s socialization to gender roles. Adolescence.

[33] Trost S.G., Pate R.R., Sallis J.F., Freedson P.S., Taylor W.C., Dowda M., Sirard J. (2002). Age and gender differences in objectively measured physical activity in youth. Med. Sci. Sports Exerc..

[34] Gray C., Gibbons R., Larouche R., Sandseter E.B., Bienenstock A., Brussoni M., Chabot G., Herrington S., Janssen I., Pickett W. (2015). What is the relationship between outdoor time and physical activity, sedentary behaviour, and physical fitness in children? A systematic review. Int. J. Environ. Res. Public Health.

[35] Brussoni M., Gibbons R., Gray C., Ishikawa T., Sandseter E., Bienenstock A., Chabot G., Fuselli P., Herrington S., Janssen I. (2015). What is the relationship between risky outdoor play and health in children? A systematic review. Int. J. Environ. Res. Public Health.

[36] Truelove S., Bruijns B.A., Vanderloo L.M., O’Brien K.T., Johnson A.M., Tucker P. (2018). Physical activity and sedentary time during childcare outdoor play sessions: A systematic review and meta-analysis. Prev. Med..

[37] Moher D., Liberati A., Tetzlaff J., Altman D.G. (2009). Preferred reporting items for systematic reviews and meta-analyses: The PRISMA statement. Ann. Intern. Med..

[38] Santos C.M.D.C., Pimenta C.A.D.M., Nobre M.R.C. (2007). The PICO strategy for the research question construction and evidence search. RLAE.

[39] Xu H., Wen L.M., Rissel C. (2015). Associations of parental influences with physical activity and screen time among young children: A systematic review. J. Obes..

[40] Sallis J.F., Prochaska J.J., Taylor W.C. (2000). A review of correlates of physical activity of children and adolescents. Med. Sci. Sports Exerc..

[41] Downes M.J., Brennan M.L., Williams H.C., Dean R.S. (2016). Development of a critical appraisal tool to assess the quality of cross-sectional studies (AXIS). BMJ Open.

[42] Schoeppe S., Duncan M.J., Badland H., Oliver M., Curtis C. (2013). Associations of children’s independent mobility and active travel with physical activity, sedentary behaviour and weight status: A systematic review. Med. Sci. Sports Exerc..

[43] Bates B., Stone M.R. (2015). Measures of outdoor play and independent mobility in children and youth: A methodological review. Med. Sci. Sports Exerc..

[44] Newell S.A., Bowman J.A., Cockburn J.D. (2000). Can compliance with nonpharmacologic treatments for cardiovascular disease be improved?. Am. J. Prev. Med..

[45] Arango C.M., Páez D.C., Reis R.S., Brownson R.C., Parra D.C. (2013). Association between the perceived environment and physical activity among adults in Latin America: A systematic review. Int. J. Behav. Nutr. Phys. Act..

[46] Van Der Horst K., Oenema A., Ferreira I., Wendel-Vos W., Giskes K., van Lenthe F., Brug J. (2006). A systematic review of environmental correlates of obesity-related dietary behaviors in youth. Health Educ. Res..

[47] Ferreira I., van der Horst K., Wendel-Vos W., Kremers S., van Lenthe F.J., Brug J. (2007). Environmental correlates of physical activity in youth—A review and update. Obes. Rev..

[48] Nicksic N.E., Salahuddin M., Butte N.F., Hoelscher D.M. (2018). Associations between parent-perceived neighborhood safety and encouragement and child outdoor physical activity among low-income children. J. Phys. Act. Health.

[49] Schoeppe S., Duncan M.J., Badland H.M., Oliver M., Browne M. (2014). Associations between children’s independent mobility and physical activity. BMC Public Health.

[50] Lubans D.R., Boreham C.A., Kelly P., Foster C.E. (2011). The relationship between active travel to school and health-related fitness in children and adolescents: A systematic review. Int. J. Behav. Nutr. Phys. Act..

[51] Bagordo F., De Donno A., Grassi T., Guido M., Devoti G., Ceretti E., Zani C., Feretti D., Villarini M., Moretti M. (2017). Lifestyles and socio-cultural factors among children aged 6–8 years from five Italian towns: The MAPEC_LIFE study cohort. BMC Public Health.

[52] Loucaides C.A., Tsangaridou N. (2017). Associations between parental and friend social support and children’s physical activity and time spent outside playing. Int. J. Pediatr..

[53] McFarland A.L., Zajicek J.M., Wallczek T.M. (2014). The relationship between parental attitudes toward nature and the amount of time children spend in outdoor recreation. J. Leis. Res..

[54] McHale S.M., Crouter A.C., Tucker C.J. (2001). Free-time activities in middle childhood: Links with adjustment in early adolescence. Child Dev..

[55] Tandon P.S., Zhou C., Christakis D.A. (2012). Frequency of parent-supervised outdoor play of us preschool-aged children. Arch. Pediatr. Adolesc. Med..

[56] Aarts M.J., Wendel-Vos W., van Oers H.A.M., van de Goor I.A.M., Schuit A.J. (2010). Environmental determinants of outdoor play in children a large-scale cross-sectional study. Am. J. Prev. Med..

[57] Wilkie H.J., Standage M., Gillison F.B., Cumming S.P., Katzmarzyk P.T. (2018). The home electronic media environment and parental safety concerns: Relationships with outdoor time after school and over the weekend among 9–11 year old children. BMC Public Health.

[58] Aarts M.J., de Vries S.I., van Oers H.A.M., Schuit A.J. (2012). Outdoor play among children in relation to neighborhood characteristics: A cross-sectional neighborhood observation study. Int. J. Behav. Nutr. Phys. Act..

[59] Bringolf-Isler B., Grize L., Mader U., Ruch N., Sennhauser F.H., Braun-Fahrlander C., Team S. (2010). Built environment, parents’ perception, and children’s vigorous outdoor play. Prev. Med..

[60] Burdette H.L., Whitaker R.C. (2005). A national study of neighborhood safety, outdoor play, television viewing, and obesity in preschool children. Pediatrics.

[61] Carsley S., Liang L.Y., Chen Y., Parkin P., Maguire J., Birken C.S., Collaboration T.A.K. (2016). The impact of daycare attendance on outdoor free play in young children. J. Public Health.

[62] Ferrao T., Janssen I. (2015). Parental encouragement is positively associated with outdoor active play outside of school hours among 7–12 year olds. PeerJ.

[63] Kimbro R.T., Brooks-Gunn J., McLanahan S. (2011). Young children in urban areas: Links among neighborhood characteristics, weight status, outdoor play, and television watching. Soc. Sci. Med..

[64] Marino A.J., Fletcher E.N., Whitaker R.C., Anderson S.E. (2012). Amount and environmental predictors of outdoor playtime at home and school: A cross-sectional analysis of a national sample of preschool-aged children attending Head Start. Health Place.

[65] McDonald S., Dowda M., Colabianchi N., Porter D., Dishman R.K., Pate R.R. (2015). Perceptions of the neighborhood environment and children’s afterschool moderate-to-vigorous physical activity. Pediatr. Excer. Sci..

[66] Wijtzes A.I., Jansen W., Bouthoorn S.H., Pot N., Hofman A., Jaddoe V.W.V., Raat H. (2014). Social inequalities in young children’s sports participation and outdoor play. Int. J. Behav. Nutr. Phys. Act..

[67] Xu H., Wen L.M., Rissel C. (2014). Associations of maternal influences with outdoor play and screen time of two-year-olds: Findings from the Healthy Beginnings Trial. J. Paediatr. Child Health.

[68] Remmers T., Broeren S.M.L., Renders C.M., Hirasing R.A., van Grieken A., Raat H. (2014). A longitudinal study of children’s outside play using family environment and perceived physical environment as predictors. Int. J. Behav. Nutr. Phys. Act..

[69] Remmers T., Van Kann D., Gubbels J., Schmidt S., de Vries S., Ettema D., Kremers S.P.J., Thijs C. (2014). Moderators of the longitudinal relationship between the perceived physical environment and outside play in children: The KOALA birth cohort study. Int. J. Behav. Nutr. Phys. Act..

[70] Xu H., Wen L.M., Hardy L.L., Rissel C. (2016). A 5-year longitudinal analysis of modifiable predictors for outdoor play and screen-time of 2- to 5-year-olds. Int. J. Behav. Nutr. Phys. Act..

[71] Veitch J., Salmon J., Ball K. (2009). The validity and reliability of an instrument to assess children’s outdoor play in various locations. J. Sci. Med. Sport.

[72] George D., Mallery P. (2016). IBM SPSS Statistics 23 Step by Step: A Simple Guide and Reference.

[73] Eyler A.E., Wilcox S., Matson-Koffman D., Evenson K.R., Sanderson B., Thompson J., Wilbur J., Rohm-Young D. (2002). Correlates of physical activity among women from diverse racial/ethnic groups. J. Women’s Health Gend Based Med..

[74] Wang S., Ramsden M. (2018). Revisiting the “parallel lives” thesis: Neighbourhood attachment and residential integration of ethnic minorities in England. Popul. Space Place.

[75] Kimbro R.T., Schachter A. (2011). Neighborhood poverty and maternal fears of children’s outdoor play. Fam. Relat..

[76] Hilbrecht M., Shaw S.M., Johnson L.C., Andrey J. (2008). ‘I’m home for the kids’: Contradictory implications for work–life balance of teleworking mothers. Gend. Work Organ..

[77] West J. (1995). Child Care and Early Education Program Participation of Infants, Toddlers, and Preschoolers. Statistics in Brief.

[78] Finn K., Johannsen N., Specker B. (2002). Factors associated with physical activity in preschool children. J. Pediatr..

[79] Chesley N. (2011). Stay-at-home fathers and breadwinning mothers: Gender, couple dynamics, and social change. Gend. Soc..

[80] Kawachi I., Berkman L. (2000). Social cohesion, social capital, and health. Soc. Epidemiol..

[81] Coleman J.S. (1988). Social capital in the creation of human capital. Am. J. Soc..

[82] Kawachi I., Kennedy B.P., Glass R. (1999). Social capital and self-rated health: A contextual analysis. Am. J. Public Health.

[83] Brockman R., Jago R., Fox K.R., Thompson J.L., Cartwright K., Page A.S. (2009). “Get off the sofa and go and play”: Family and socioeconomic influences on the physical activity of 10–11 year old children. BMC Public Health.

[84] Watchman T., Spencer-Cavaliere N. (2017). Times have changed: Parent perspectives on children’s free play and sport. Psychol. Sport Exerc..

[85] Refshauge A.D., Stigsdotter U.K., Cosco N.G. (2012). Adults’ motivation for bringing their children to park playgrounds. Urban For. Urban Green..

[86] Ross C.E., Jang S.J. (2000). Neighborhood disorder, fear, and mistrust: The buffering role of social ties with neighbors. Am. J. Commun. Psychol..

[87] Ceballo R., McLoyd V.C. (2002). Social support and parenting in poor, dangerous neighborhoods. Child Dev..

[88] Morrongiello B.A., Dawber T. (1999). Parental influences on toddlers’ injury-risk behaviors: Are sons and daughters socialized differently?. J. Appl. Dev. Psychol..

[89] Tranter P., Pawson E. (2001). Children’s access to local environments: A case-study of Christchurch, New Zealand. Local Environ..

[90] Little H., Wyver S. (2010). Individual differences in children’s risk perception and appraisals in outdoor play environments. Int. J. Early Years Educ..

[91] Eaton W.O., Enns L.R. (1986). Sex differences in human motor activity level. Psychol. Bull..

[92] Beets M.W., Vogel R., Forlaw L., Pitetti K.H., Cardinal B.J. (2006). Social support and youth physical activity: The role of provider and type. Am. J. Health Behav..

