# Supplementary material for: Parental Correlates of Outdoor Play in Boys and Girls Aged 0 to 12—A Systematic Review
Source: Int J Environ Res Public Health. 2019 Jan 11;16(2):190. doi: 10.3390/ijerph16020190 (PMC6351982; doi:10.3390/ijerph16020190)
Supplement: Supplementary file 1 [file ijerph-16-00190-s001.zip › ijerph-397145 Supplementary - For Proof/ijerph-397145 Table S1.pdf]

Table S1. Extracted data of studies included in systematic review (multivariate findings).

#	Author (year)<br>Country	Study design / Data collection / Participants (sample size; age range (mean); gender; response rate)	Outdoor Play<br>Definition<br>Measurement	Parental correlates<br>Measurement	Multivariate findings	Study quality
1	**Aarts et al. (2010) [56]**<br><br>Netherlands	<u>**Study design**</u><br>cross-sectional<br><br><u>**Data collection**</u><br>Sep 2007 - Jan 2008<br><br><u>**Participants**</u><br>n = 6,470<br>4-12 yrs (*7.9 yrs*)<br>*49.9% (F)*<br>rr = 60%	<u>**Definition**</u><br>natural way for children to be physically active [56]; in contrast to organized sports, outdoor play is cheap, informal and easily accessible [8]<br><br><u>**Measurement**</u><br>**parental report:** (considering a typical week in the last mo./separately questions for week- and week-end days): *'How many days does your child play outside on week-days/weekends?'*; *'On average, how long does your child spend*	<u>**Socio-demographic and biological correlates**</u><br>**(1)** parental education level<br><br><u>**Parents' psychological, cognitive and emotional correlates**</u><br>**(2)** importance of outdoor play<br><br><u>**Parenting practices**</u><br>**(3)** presence of rules<br><br><u>**Parents' perceived neighborhood physical environment**</u><br>**(4)** satisfaction with play facilities<br>**(5)** satisfaction with public and green space<br>**(6)** degree of unoccupied houses<br>**(7)** presence of trash and litter<br>**(8)** presence of dog waste<br>**(9)** degree of high-rise buildings<br>**(10)** presence of green in the neighborhood<br>**(11)** presence of water<br>**(12)** traffic situation	**Multivariate GEE analysis** (Relative rate; 95% CI):<br>- adjusted for parental education<br><br><u>**Socio-demographic and biological correlates**</u><br>**(1)** parental education level is negatively associated (RR range = 0.93-0.97, CI range: 0.90, 0.98) with outdoor play in children (4-12 yrs)<br><br><u>**Parents' psychological, cognitive and emotional correlates**</u><br>**(2)** importance parents place to outdoor play is positively associated (RR range = 1.32-1.75, CI range: 1.09, 2.16) with outdoor play in children (4-12 yrs)<br><br><u>**Parenting practices**</u><br>**(3)** presence of rules: no significantly association with outdoor play in children (4-12 yrs)<br><br><u>**Parents' perceived neighborhood physical environment**</u>	***high***

	on outdoor play on such a weekday/ weekend day?' [93]	**(13)** quality of sidewalks and bike lanes<br>**(14)** diversity of routes<br>**(15)** distance to facilities<br><br>**<u>Parents' perceived neighborhood social environment</u>**<br>**(16)** social safety<br>**(17)** social cohesion<br>**(18)** satisfaction with social contacts<br><br>**<u>Measurements</u>**<br>**parental report:** (2) – (18) response options: Five-point Likert scale from 'strongly agree' to 'strongly disagree' [93]	**(4)** no significant association of the satisfaction with play facilities and outdoor play in children (4-12 yrs)<br><br>**(5)** no significant association of the satisfaction with public space and green space and outdoor play in children (4-12 yrs)<br><br>**(6)** no significant association of the degree of unoccupied houses and outdoor play in children (4-9 yrs)<br><br>**(7)** no significant association of the presence of trash and litter and outdoor play in children (4-12 yrs)<br><br>**(8)** no significant association of the presence of dog waste and outdoor play in children (7-12 yrs)<br><br>**(9)** no significant association of the degree of high-rise buildings and outdoor play in children (4-12 yrs)<br><br>**(10)** no significant association of the presence of green in the neighborhood and outdoor play in children (4-12 yrs)<br><br>**(11)** no significant association of the presence of water and outdoor play in children (7-12 yrs)<br><br>**(12)** no significant association of the traffic situation and outdoor play in children (4-12 yrs)

					**(13)** no significant association of the perceived quality of sidewalks and bike lanes and outdoor play in children (4-12 yrs)<br><br>**(14)** no significant association of the perceived diversity of routes and outdoor play in children (4-6 yrs)<br><br>**(15)** no significant association of the perceived distance to facilities and outdoor play in children (4-12 yrs)<br><br>**Parents' perceived neighborhood social environment**<br>**(16)** social safety is positively associated with outdoor play in boys (RR =1.02, CI: 1.01, 1.03) and girls (RR = 1.01, CI: 1.00, 1.03) (4-6 yrs); no significance for boys and girls (7-9 and 10-12 yrs)<br><br>**(17)** perceived social cohesion is positively associated with outdoor play in children (4-6 and 7-9 yrs; RR range = 1.01-1.02, CI: 1.00, 1.03)<br><br>**(18)** no significant association of the satisfaction with social contacts and outdoor play in children (4-12 yrs)	
**2**	**Aarts et al. (2012) [58]**<br><br>Netherlands	**Study design**<br>cross-sectional<br><br>**Data collection**<br>characteristics	**Definition**<br>way to stimulate physical activity in children, by	**Socio-demographic and biological correlates**<br>**(1)** parental education level<br><br>**Measurements**	**Multivariate regression analysis** (Relative rate; 95% CI):<br>- adjusted for child's age, parental education level, environmental characteristics	***high***

		of participants<br>Sep 2007 – Jan 2008;<br>neighborhood characteristics<br>Oct - Dec 2008<br><br>**<u>Participants</u>**<br>n = 3,651<br>4-12 yrs (7.8 yrs)<br>*49.4% (F)*<br>rr = 60%	stimulating outdoor play [8]<br><br>**<u>Measurement parental report</u>**:<br>'*number of school days and number of days per week-end in a typical week in the past mo.*'; '*outdoor play duration (response options: less than 30 min/ day; 30 min to one hr/day; one to two hr/day; more than two hr/day)*	**parental report**:<br>**(1)** indicator for socio-economic status in The Netherlands [94]	**<u>Socio-demographic and biological correlates</u>**<br>**(1)** higher parental education level is negatively associated (RR range = 0.94-0.96, CI: 0.91, 1.00) with outdoor play in children (7-12 yrs); no significant association for 4-6 yrs old children	
**3**	**Bringolf-Isler et al. (2010) [59]**<br><br>Switzerland	**<u>Study design</u>**<br>cross-sectional<br><br>**<u>Data collection</u>**<br>Aug 2004 - Jun 2005<br><br>**<u>Participants</u>**<br>n = 680<br>6/7 and 9/10 yrs (n.c.)<br>48.8% (F)<br>rr = 65%	**<u>Definition</u>**<br>a proposed and validated measure of PA in children [95-97]<br><br>**<u>Measurement</u>**<br>**parental report:**<br>parent's indication *how much time their child spent on average vigorously playing outdoors on weekdays and weekends* (response options: 'zero min;	**<u>Socio-demographic and biological correlates</u>**<br>**(1)** maternal education level<br><br>**<u>Parents' perceived neighborhood physical environment</u>**<br>**(2)** perceptions of 'traffic problems'<br>**(3)** availability of garden or park<br>**(4)** playing in neighbors garden<br>**(5)** playing in fields (further away than 500 m)<br><br>**<u>Parents' perceived neighborhood social environment</u>**<br>**(6)** perceptions of crime problems	**Multivariate linear regression** (adjusted mean; 95% CI; p-value):<br>- adjusted for socio-cultural factors<br><br>**<u>Socio-demographic and biological correlates</u>**<br>**(1)** no association of maternal education and outdoor play in children (p>0.05)<br><br>**<u>Parents' perceived neighborhood physical environment</u>**<br>**(2)** children of parents who perceived traffic problems (70.3; 58.2, 82.4; p=0.01) spent significantly less time outdoors (Ref. parents without perception of traffic problems)	*fair*

			<30 min; 30 min to one hr; one hr to two hrs; two to four hrs; and >4 hrs')	**Measurements**<br>**parental report:**<br>**(1)** response options: '<2 yrs of professional training'; medium: '2-4 yrs of professional training'; high: 'college or university education'<br><br>**(3) – (6)** if there is a problem; which problem prevent their child from playing outdoors (response options: 'traffic', 'no garden/green space available', or 'crime')	**(3)** children of parents who perceived problems of nonavailability of garden **and** park (63.6; CI: 39.3, 87.8; p=0.09) spent significantly less time outdoors (Ref. parents without perceptions of nonavailability of garden **and** park); no association perceived problems of nonavailability of garden **or** park<br><br>**(4)** children of parents who perceived that child plays in neighborhoods garden (85.3; CI: 79.8, 90.9; p=0.003) spent significantly more time outdoors<br><br>**(5)** children of parents who perceived that child plays in fields and woods further away than 500m (92.5; CI: 85.0, 100.0; p=<0.001) spent significantly more time outdoors<br><br>**Parents' perceived neighborhood social environment**<br>**(6)** children of parents who perceived problems of crime (63.5; CI: 35.5, 91.5; p=0.1) spent significantly less time outdoors (Ref. parents without perception of problems of crime)	
**4**	**Burdette et al. (2005) [60]**<br><br>United States	**Study design**<br>cross-sectional<br><br>**Data collection**<br>birth cohort 1998-2000 (The Fragile Families and Child	**Definition**<br>n.r.<br><br>**Measurement**<br>**parental report:**<br>*number of hrs child 'typically' spends playing outdoors each day* (separately for	**Parents' perceived neighborhood social environment**<br>**(1)** maternal perceptions of neighborhood safety<br><br>**Measurements**<br>**parental report:**<br>**(1)** Neighborhood Environment for Children Rating Scales [98]	**General linear models** (p-value):<br>- adjusted for household income, mothers' education, race/ethnicity, child's age, marital status<br><br>**Parents' perceived neighborhood social environment**<br>**(1)** no association of maternal perceptions of neighborhood safety and outdoor play in	*fair*

		Wellbeing Study)<br><br>**Participants**<br>n = 3,141<br>n.m. (*3.25 yrs*)<br>47% (F)<br>rr = n.r.	week- and weekend days)		children for weekdays (p= 0.24) and for weekend days (p= 0.16)	
**5**	**Carsley et al. (2016) [61]**<br><br>Canada	**Study design**<br>cross-sectional<br><br>**Data collection**<br>Dec 2008 - Aug 2011 (from The Applied Research Group for Kids - TARGet Kids!)<br><br>**Participants**<br>n = 2,810<br>1-5 yrs (n.c.)<br>48.4% (F)<br>rr = n.r.	**Definition**<br>active free play is a way to occur physical activity in young children; also referred as active outdoor play, active play, unstructured free play and physically active play [99,100]; occurs usually outdoors and has been associated with increased moderate- to vigorous-intensity physical activity (MVPA) [100]<br><br>**Measurement**<br>**parental report**:<br>*'Aside from time in daycare and pre-school, on a typical*	**Socio-demographic and biological correlates**<br>**(1)** maternal ethnicity<br>**(2)** household income<br>**(3)** maternal education level<br><br>**Measurements**<br>**parental report**	**Multivariate linear regression** (adjusted β coefficient; 95% CI; p-value):<br>- adjusted for child's age, child's sex, maternal ethnicity, maternal education, neighborhood income, season<br><br>**Socio-demographic and biological correlates**<br>**(1)** no association of maternal ethnicity (p>0.05) and outdoor play in children (1-<3 yrs); negative association of maternal East Asian ethnicity (β=-10.1; -17.5,-2.8; p<0.01) and outdoor play in children (≥3-5 yrs); negative association of maternal South and Southeast Asian ethnicity (β=-9.4; -16.7,-2.1; p=0.01) and outdoor play in children (≥3-5 yrs); negative association of maternal Other ethnicity (β=-6.1; -13.3,1.2; p=0.10) and outdoor play in children (≥3-5 yrs) (ref Caucasian ethnicity)<br><br>**(2)** no association of household income (p>0.05) and outdoor play in children (1-5 yrs)<br><br>**(3)** no association of maternal education level (p>0.05) and outdoor play in children (1-5 yrs)	*fair*

			weekday, how much time does your child spend outside in "unstrucktured free play"?' (response in min/day)			
**6**	**Cleland et al. (2010) [23]**<br><br>Australiare	**Study design**<br>longitudinal<br><br>**Data collection**<br>Jul - Dec 2001, 2004 and 2006 (The Children Living in Active Neighborhoods (CLAN) study)<br><br>**Participants**<br>n = 421<br>5-6 and 10-12 yrs at baseline (n.c.)<br>n.c. (F)<br>rr = 38.3%	**Definition**<br>n.r.<br><br>**Measurement parental report:**<br>*'In total, how many hrs/min does your child usually spend outside during a typical week after school?'* (separately for week- and weekend days)	**Parenting practices**<br>**(1)** no adults to supervise child's outdoor play<br><br>**Parents' social and cultural correlates**<br>**(2)** parental encouragement<br>**(3)** outdoor social opportunities<br><br>**Measurements parental report:**<br>**(1)** responses to two statements about encouragement of and success in getting the child to play outside (ICC 0.42-0.54)<br><br>**(2)** four statements about who the child goes outside with (ICC 0.61-0.79)<br><br>**(3)** reported agreement on a five-point Likert scale: 'I don't allow my child to play outside after dark.; I don't allow my child to walk/ride a bike on the street after dark.' (ICC 0.21-0.36)	**Longitudinal linear regression** (adjusted β coefficient; 95% CI; p-value):<br>- adjusted for maternal education, parental marital status, outdoor tendencies, parental encouragement, social opportunities, supervision, dog ownership, yard size, home PA opportunities, weather<br><br>**Parenting practices**<br>**(1)** no association of adults supervision and time spent outdoors for boys and girls (5-6 yrs)<br><br>**(1)** no adults for supervise children's outdoor play is negatively associated with average weekly time (minutes/week) spent outdoors in boys (β=-46.9; CI: -91.3, -2.6; $p<0.05$) and girls (β=-34.0; CI: -59.6, -9.1; $p<0.01$) (10-12 yrs)<br><br>**Parents' social and cultural correlates**<br>**(2)** no significant association of medium parental encouragement and outdoor play in boys and girls (5-6 to 10-12 yrs)<br><br>**(3)** no significant association of medium social opportunities with average weekly time (minutes/week) spent outdoors in boys and girls (5-6 to 10-12 yrs) (ref low) ($p>0.05$)	***fair***

					**(3)** no association of high social opportunities and time spent outdoors in boys and girls (10-12 yrs)	
**7**	**Faulkner et al. (2015) [5]**<br><br>Canada	<u>**Study design**</u><br>cross-sectional<br><br><u>**Data collection**</u><br>Apr 2010 - May 2011 (from Project BEAT: Built Environment and Active Transport)<br><br><u>**Participants**</u><br>n = 859<br>grades 5-6<br>(10.50 yrs)<br>52.5% (F)<br>rr = 84%	<u>**Definition**</u><br>Free play may be the most natural and effective form of learning and is vital for children's happiness [101]; Active play involves physical activity at energy costs above resting levels but below 'exercise' levels [102]<br><br><u>**Measurement**</u><br>**parental report:**<br>'*On a typical weekday/weekend day, how much time does your child spend playing outdoors?*' (separately for week- and weekend days - response options: 'None' to 'More than 3 hrs' (7-point Likert scale))	<u>**Socio-demographic and biological correlates**</u><br>**(1)** parental employment status<br><br><u>**Parents' perceived neighborhood physical environment**</u><br>**(2)** not enough sidewalks<br>**(3)** not enough attractive roads for child to walk<br>**(4)** drivers go to fast (in the neighborhood)<br><br><u>**Parents' perceived neighborhood social environment**</u><br>**(6)** worry about children's interactions with strangers<br>**(7)** people are out and about, talking, and doing things with one another<br>**(8)** living in a safe neighborhood<br><br><u>**Measurements**</u><br>**parental report:**<br>**(1)** part-time worker/ homemaker; full-time worker<br><br>**(2) – (8**) 5-point Likert scales ('Strongly agree' to 'Strongly disagree')	**Adjusted ordered logit model** (β coefficient; p-value):<br>- adjusted for 6 items of perceived physical environment<br><br><u>**Socio-demographic and biological correlates**</u><br>**(1)** positive association of parental employment status (part-time worker/homemaker) (β=0.42; p= 0.002) and outdoor play in children for weekend days; no association for weekdays<br><br><u>**Parents' perceived neighborhood physical environment**</u><br>**(2)** no significant association of sufficiency of sidewalks and outdoor play in children (p>0.05) for week- and weekend days<br><br>**(3)** no association of roads attractiveness and outdoor play in children (p>0.05) for week-and weekend days<br><br>**(4)** negative association of fast driving in neighborhood (β=-0.30; p = 0.025) and outdoor play in children for weekdays; no significant association for weekend days (p>0.05)<br><br><u>**Parents' perceived neighborhood social environment**</u>	*fair*

					**(6)** negative association of perceptions of children's interactions with strangers and outdoor play for week- (β=-0.32; p= 0.019) and weekend days (β=-0.35; p = 0.010)<br><br>**(7)** positive association of perception of people interactions and outdoor play in children for week- (β=0.56; p>0.000) and weekend days (β=0.57; p>0.000)<br><br>**(8)** no association of neighborhood safety and outdoor play in children (p>0.05) for week- and weekend days	
**8**	**Ferrao & Janssen (2015) [62]**<br><br>United States	**<u>Study design</u>**<br>cross-sectional<br><br>**<u>Data collection</u>**<br>Nov 24th 2014 from births 2003 – 2007<br><br>**<u>Participants</u>**<br>n = 514<br>7-12 yrs (n.c.)<br>51.2% (F)<br>rr = n.r.	**<u>Definition</u>**<br>Active play (e.g., tag, road hockey, hide and seek) is one of four primary domains in which children accumulate their moderate-to-vigorous physical activity (MVPA) [103]<br><br>**<u>Measurement</u>**<br>**parental report:**<br>'*Thinking about the past month, in a usual week how often did your child play outdoors in the*	**<u>Socio-demographic and biological correlates</u>**<br>**(1)** family structure<br>**(2)** household income<br>**(3)** parental education level<br><br>**<u>Parenting practices</u>**<br>**(4)** permission for independent mobility<br><br>**<u>Parents' social and cultural correlates</u>**<br>**(5)** parental encouragement<br>**(6)** parental facilitation<br>**(7)** parental involvement<br>**(8)** parental modelling<br><br>**<u>Parents' perceived neighborhood physical environment</u>**<br>**(9)** perceptions of unsafe roads<br>**(10)** perceptions of heavy traffic	**Multivariate models** (β coefficient; S.E.):<br>- adjusted for gender, age, race, parental structure, number of siblings in household, household income, parental education, independent mobility, parental facilitation score, parental involvement score, parental modeling score, population size of municipality, urban form, unsafe roads factor, traffic calming factor, safe for children factor, crime risk factor<br><br>**<u>Socio-demographic and biological correlates</u>**<br>**(1) - (3)** no significant association of parental structure; household income; parental education and outdoor play frequency in children<br><br>**<u>Parenting practices</u>**<br>**(4)** no significant association of permission for independent mobility and outdoor play frequency in children	*fair*

	following locations? (yard at home, yard of someone else's home, street or cul-de-sac of your home, other streets or cul-de-sacs, parks and play-grounds outside of school hours, school grounds outside of school hours, other places where the child can be active) (ICC = 0.58-0.82) [71]	**Parents' perceived neighborhood social environment**<br>**(11)** perceptions outdoor play is safe for children<br>**(12)** perceptions of crime risks<br><br>**Measurements**<br>**parental report:**<br>**(1)** two parents; single parent<br><br>**(3)** high school or less; 2 year college; 4 year college/university; graduate university or higher<br><br>**(4)** permission for independent mobility: response options range: from 'my child is not allowed out alone' to 'my child is allowed out more than a 15 min walk from home' [104]<br><br>**(5)** response to statements: i.e. 'I encourage my child to use resources in our neighborhood to be active'; 'I encourage my child to play outdoors (without adult supervision) when the weather is nice' (response options: 'never/rarely (0)'; 'less than once a week (0.5)'; '1-2 times per week (1.5)'; '3-4 times per week' (3.5); '5-6 times per week' (5.5); 'daily') (7); average range of points assigned in quintiles: Q1: 0-1.4; Q2: 1.5-2.5; Q3: 2.6-3.6; Q4: 3.8-5; Q5: 5.3-7 [105]	**Parents' social and cultural correlates**<br>**(6)** significant increase in parental encouragement (β range= 2.5-12.7; S.E. range= 1.1-1.2) is associated with higher outdoor play frequency in children<br><br>**(6)** and **(8)** no significant association of parental facilitation; parental modelling and outdoor play frequency in children<br><br>**(7)** increasing in parental involvement score (β=1.1; S.E.=0.5) is associated with higher outdoor play frequency score in children<br><br>**Parents' perceived neighborhood physical environment**<br>**(9)** and **(10)** increasing in unsafe roads factor (β=0.9; S.E.=0.4); traffic calming factor (β=1.0; S.E.=0.4) is associated with higher outdoor play frequency score in children<br><br>**Parents' perceived neighborhood social environment**<br>**(11)** and **(12)** increasing in safe for children factor (β=3.1; S.E.=0.4); crime risk factor (β=1.7; S.E.=0.4) is associated with higher outdoor play frequency score in children

				**(6) – (8)** items from Activity Support Scale for Multiple Groups (ACTS-MG) [105]<br><br>**(9) - (12)** unsafe roads, traffic calming, safe for children, crime risk [106]		
**9**	**Janssen (2015) [27]**<br><br>Canada & United States	**Study design**<br>cross-sectional<br><br>**Data collection**<br>May 13th - May 15th 2014 (from FluidSurvey™ panel)<br><br>**Participants**<br>n = 724<br>7-12 yrs (n.c.)<br>49.5% (F)<br>*rr = 42%*	**Definition**<br>n.r.<br><br>**Measurement**<br>**parental report:**<br>*'Thinking about the past mo., in a usual week how often did your child play outdoors in the following locations? (yard at home, yard of someone else's home, street or cul-de-sac of your home, other streets or cul-de-sacs, parks and play-grounds outside of school hours, school grounds outside of school hours, other places where the child can be active)* (Cronbach's α = 0.58-0.82) [71]	**Parenting practices**<br>**(1)** helicopter parenting<br>**(2)** little emperor parenting<br>**(3)** tiger mom parenting<br>**(4)** concerted cultivation parenting<br>**(5)** additive hyper parenting (score of all parenting styles)<br><br>**Measurements**<br>**parental report:**<br>20 items to assess parenting styles [107]<br><br>**(1)** i.e. 'If my child has a problem, I will come to the rescue to solve it for them.'; 'I keep tabs on my child's activities.'; 'I expect to be involved in my child's decisions about how to spend their time and money.' (Cronbach's α = 0.64)<br><br>**(2)** i.e. 'My household structures itself around the desires of my child.'; 'My child commands the sole attention of their parents or guardians.' (Cronbach's α = 0.80)<br><br>**(3)** i.e. 'I expect the best from my child and don't settle for anything less.'; 'I don't believe in making exceptions or excuses	**Multivariate models** (mean (range); 95% CI (range); p-value):<br>- adjusted for gender, age, race, parental structure, number of children in household, household income, parental education, rural/urban status<br><br>**Parenting practices**<br>**(1)** no association of helicopter parenting groups (13.0-14.5; CI: 10.7, 16.3; p>0.05) and outdoor play in children<br><br>**(2)** positive association of low 'little emperor parenting' scores (15.7; CI: 13.9, 17.5; p<0.005) (ref average, above average)<br><br>**(3) - (4)** positive association of low 'tiger mom parenting' scores (16.4; CI: 14.5, 18.3; p<0.050); low 'concerned cultivation parenting' scores (17.2; CI: 15.3, 19.1; p<0.005) and outdoor play in children (ref average, above average and high scores)<br><br>**(5)** positive association of additive low hyper-parenting score (20.2; CI: 17.5, 23.0; p<0.005) and outdoor play in children	***high***

				for my child's failures.' (Cronbach's α = 0.60)<br><br>**(4)** i.e. 'My child participates in more extra-curricular activities than other children.'; 'My child has little free time to play with friends.' (Cronbach's α = 0.62)		
10	**Kimbro et al. (2011) [63]**<br><br>United States	**Study design**<br>cross-sectional<br><br>**Data collection**<br>birth cohort 1998 and 2000; follow up at year 1, year 3 and year 5 (from Fragile Families and Child Wellbeing Survey)<br><br>**Participants**<br>n = 1,822<br>5 yrs (*5.29 yrs*)<br>49% (F)<br>rr = 76%	**Definition**<br>n.r.<br><br>**Measurement**<br>**maternal report:**<br>*child's average number of hrs/weekday of outdoor play*	**Socio-demographic and biological correlates**<br>**(1)** maternal education level<br>**(2)** maternal employment status<br>**(3)** family structure<br>**(4)** maternal age<br>**(5)** household income<br>**(6)** maternal body mass index (BMI)<br><br>**Parents' psychological, cognitive and emotional correlates**<br>**(7)** maternal depression<br>**(8)** concerns let the child play outside<br><br>**Measurements**<br>**maternal report:**<br>**(1)** 'did not complete high school'; 'completed high school'; 'some college or more'<br><br>**(2)** 'not employed outside the home'; 'full-time'; 'part-time' work<br><br>**(3)** 'married with child's biological or social father'; cohabiting; single	**Multivariate models** (β coefficient (Exp. Coeff.); p-value):<br>- adjusted for clustering at the city-level<br><br>**Socio-demographic and biological correlates**<br>**(1)**, **(4) - (6)** no association of maternal education level; mother's age; household income; mother's BMI (p>0.05) and outdoor play in children<br><br>**(2)** negative association of mother's employment status: full-time (β =-0.12 (0.89); p<0.001); part-time (β =-0.14 (0.87); p<0.05) and outdoor play in children (Ref. mother does not work)<br><br>**(3)** positive association of family structure (mother is no longer with (social) father) (β=0.10 (1.10); p<0.10) and outdoor play in children (ref mother is married to father/ social father)<br><br>**Parents' psychological, cognitive and emotional correlates**<br>**(7)** no association of maternal depression (p>0.05) and outdoor play in children	***high***

				**(6)** mother's BMI (between 25.0-29.9 overweight; ≥30 obese)<br><br>**(7)** maternal depression: CIDI-SF [108]<br><br>**(8)** is mother feeling fearful about her child playing outdoors; response options: yes/no	**(8)** no association of maternal concerns to let the child play outside (p>0.05) and outdoor play in children	
11	**Marino et al. (2012) [64]**<br><br>United States	**Study design**<br>cross-sectional<br><br>**Data collection**<br>fall 2006 - spring 2007 (from Head Start Family and Child Experiences Survey (FACES) 2006)<br><br>**Participants**<br>n = 2,529<br>52.2-53.8 mos. (*4.42 yrs*)<br>48.9% (F)<br>rr = n.r.	**Definition**<br>n.r.<br><br>**Measurement**<br>**parental report:**<br>'*We are interested in the kinds of things [CHILD] did on the last day you followed your regular routine. Did your child spend any time playing outside?*'; if response was 'yes': *About how much time does [CHILD] spend playing outside on a typical weekday?* Would you say more than 2 hrs, 1–2 hrs, or less than 1 hr?'	**Parents' social and cultural correlates**<br>**(1)** playground/ park visits or have a picnic<br><br>**Measurements**<br>**parental report:**<br>**(1)** 'In the past month, has anyone in your family done the following thigs with [CHILD]? Visited a playground, park, or gone on a picnic?'	**Multivariate models** (OR; 95% CI):<br>- adjusted for child's age, gender, race/ethnicity, weight status, mother's education, single-parent household, Head Start program type, month of interview, region<br><br>**Parents' social and cultural correlates**<br>**(1)** positive association of playground/park vision or have a picnic (1.74; CI: 1.18, 2.56) and outdoor play (more than 2h/week) in children	*high*
12	**McDonald et al. (2015) [65]**	**Study design**<br>cross-sectional	**Definition**<br>n.r.	**Parents' social and cultural correlates**<br>**(1)** perceived parental support	**Structural equation model** (β coefficient; p-value):	*high*

	United States	**<u>Data collection</u>**<br>from Transitions and Activity Changes in Kids (TRACK) study<br><br>**<u>Participants</u>**<br>n = 599<br>grade 5 (10.5 yrs)<br>*54.4% (F)*<br>rr = n.r.	**<u>Measurement</u>**<br>**child's report:**<br>Physical Activity Choices (PAC) survey (*frequency of days of activities child participated during last five days*) [65]	**<u>Parents' perceived neighborhood social environment</u>**<br>**(2)** overall perceptions of neighborhood (equal with child perception of the neighborhood)<br><br>**<u>Measurements</u>**<br>**parental report:**<br>**(1)** 5 items (Cronbach's α= 0.89) [109]<br><br>**(2)** 3 items: safety of the neighborhood in regard to outdoor activity; whether or not parents see other children playing outside (from Amherst Health and Activity Study parent survey [109]	- adjusted for sex, race/ethnicity, education, accelerometer wear time<br><br>**<u>Parents' social and cultural correlates</u>**<br>**(1)** positive association of parental support (β=0.20; $p<0.001$) and outdoor play in children<br><br>**<u>Parents' perceived neighborhood social environment</u>**<br>**(2)** no association of overall perceived overall neighborhood environment (β=0.10; $p>0.05$) and outdoor play in children	
**13**	**Nicksic et al. (2018) [48]**<br><br>United States	**<u>Study design</u>**<br>cross-sectional<br><br>**<u>Data collection</u>**<br>2012 - 2014 (from Texas Childhood Obesity Research Demonstration (TX CORD))<br><br>**<u>Participants</u>**<br>n = 748<br>grade 5 (n.r.)<br>57.9% (F)<br>rr = ~90%	**<u>Definition</u>**<br>n.r.<br><br>**<u>Measurement</u>**<br>**parental report:**<br>*'Most of the time does your child play outdoors for at least 30 min per day? Do not count outdoor play during school hrs.'*<br><br>**child's report:** *'Last week, on what days did you play out-doors for at least 30 min or more? Do not*	**<u>Parents' social and cultural correlates</u>**<br>**(1)** parental encouragement<br><br>**<u>Parents' perceived neighborhood social environment</u>**<br>**(2)** neighborhood safety<br><br>**<u>Measurements</u>**<br>**parental report:**<br>**(1)** extent of agreement with the statement 'If the weather is good, I encourage my child to play outside.'; (Likert scale: 'strongly disagree' to 'strongly agree') [109]<br><br>**(2)** 11-item scale (Cronbach's α= 0.91); adapted from the Teen Health 2000 Study [110]	**Multivariate models** (β coefficient; p-value):<br>- adjusted for sociodemographic variables, child's weight status, parent's PA<br><br>**<u>Parents' social and cultural correlates</u>**<br>**(1)** positive association of parental encouragement and parent's reported outdoor play for boys (β=5.23; CI:2.20,12.43;$p<0.001$) and girls (β=6.07; CI:3.13,11.78;$p<0.001$)<br><br>**<u>Parents' perceived neighborhood social environment</u>**<br>**(2)** no significant association of perceived neighborhood safety and parent's reported outdoor play in children ($p>0.05$)	***high***

			count outdoor play during school hrs.'(Spearman's correlation bet-ween child's and parents report 0.168; p=0.01)			
**14**	**Remmers, Broeren et al. (2014) [68]**<br><br>Netherlands	**Study design**<br>longitudinal study<br><br>**Data collection**<br>2007 - 2010 (from "Be Active, Eat Right" study)<br><br>**Participants**<br>n = 2,007<br>5 yrs (5.75 yrs - baseline); 7 yrs (follow-up)<br>49.5% (F)<br>rr = n.r.	**Definition**<br>play behavior without any given tasks or goals; specific type of PA behavior; natural and cheap way of increasing children's PA [68]<br><br>**Measurement parental report:**<br>total *duration of unstructured outdoor play in an average week (without organized sports, school PA or active transport)*	**Socio-demographic and biological correlates**<br>**(1)** parental age<br>**(2)** parental education<br>**(3)** parental ethnicity<br>**(4)** parental BMI<br><br>**Parents' psychological, cognitive and emotional correlates**<br>**(5)** parental attitude to improve child engagement in outdoor play<br>**(6)** perceived difficulty to improve child engagement in outdoor play<br>**(7)** intention to improve child engagement in outdoor play<br>**(8)** perception of outdoor play as habit<br><br>**Parenting practices**<br>**(9)** parental supervision of outdoor play<br>**(10)** presence rules<br>**(11)** child autonomy<br><br>**Parents' social and cultural correlates**<br>**(12)** presence of active encouragement<br>**(13)** parental PA<br>**(14)** parent's partner PA	**Multivariate models at age 5** (β coefficient; 95% CI; p-value):<br>- adjusted for socio-demographic variables, family environment, perceived physical environment<br><br>**Socio-demographic and biological correlates**<br>**(1)** no significant association of parental age and outdoor play in children (5/7 yrs) ($p>0.05$)<br><br>**(2)** no significant association of parental education and outdoor play in children (5 yrs) ($p>0.05$); negative association of high parental education (β=-26.63; CI: -54.10, 0.84; $p<0.05$) and outdoor play in children (7 yrs)<br><br>**(3)** no significant association of parental ethic background and outdoor play in children (5/7 yrs) ($p>0.05$)<br><br>**(4)** positive association of parental BMI (β=1.14; CI: 0.26, 2.02; $p<0.05$) and outdoor play in children (5/7 yrs)<br><br>**Parents' psychological, cognitive and emotional correlates**	***fair***

	**<u>Parents' perceived neighborhood social environment</u>**<br>**(15)** safety perception of outdoor play during daytime<br>**(16)** safety perception of outdoor play during evenings<br>**(17)** child-friendliness of the neighborhood<br>**(18)** attractiveness of the neighborhood for children<br>**(19)** safety of outdoor play without supervision<br><br>**<u>Measurements</u>**<br>**parental report:**<br>**(5) - (19)** items of family environment: theories of social cognitive theory [111], theory of planned behavior [112], and social leaning theory [113]; items of perceived physical environment: study [114]	**(5) - (7)** negative association of parental attitude (agree) (β=-3.78; CI: -7.46, -0.10; p<0.05); perceived difficulty (β=-16.33;CI: -26.41, -6.26; p<0.05); intention to improve child engagement in outdoor play (agree) (β=-12.83; CI: 19.73, -5.92; p<0.05) and outdoor play in children (5 yrs)<br><br>**(6)** negative associations of perceived difficulty to improve child engagement in outdoor play (β=-22.11; CI: -33.41, -10.81; p<0.05 and outdoor play in children (7 yrs)<br><br>**(8)** positive association of parental perception of outdoor play as habit and outdoor play in children (5 yrs) (β=33.41; CI: 25.05, 41.77; p<0.05) and outdoor play in children (7 yrs) (β=23.99; CI: 14.61, 33.36; p<0.05)<br><br>**(5)** and **(7)** no significant associations of parental attitude (agree); intention to improve child engagement in outdoor play (agree) and outdoor play in children (7 yrs) (p>0.05)<br><br>**<u>Parenting practices</u>**<br>**(9)** no significant associations of parental monitoring (agree) and outdoor play in children (p>0.05) (5/7 yrs)<br><br>**(10)** and **(11)** positive association of presence of outdoor play rules (β=19.87; CI: 13.44, 26.30; p<0.05); child autonomy (agree) (β=11.66; CI: 3.30, 20.02; p<0.05) and outdoor play in children (5 yrs)

(10) positive associations of presence of outdoor play rules (β=16.46; CI: 9.26, 23.67; p<0.05) and outdoor play in children (7 yrs)

(11) no significant associations of child autonomy (agree) and outdoor play in children (7 yrs) (p>0.05)

<u>Parents' social and cultural correlates</u>
(12) - **(14)** no significant associations of presence of active encouragement; PA of parent and parent's partner and outdoor play in children (5 yrs) (p>0.05)

(12) negative association of presence of active encouragement (β=-8.91; CI: -17.33, -0.48; p<0.05) and outdoor play in children (7 yrs)

(13) no significant associations of parent's PA and outdoor play in children (7 yrs) (p>0.05)

(14) positive association of parent's partner PA (β=1.85; CI: 0.27, 3.42; p<0.05) and outdoor play in children (7 yrs)

<u>Parents' perceived neighborhood social environment</u>
(15) - (17) no significant associations of safety perception during daytime and evenings; friendliness and attractiveness for children; safety of outside play without supervision and outdoor play in children (5/7 yrs) (p>0.05)

Linear mixed models for ages 5 to 7 (β coefficient; 95% CI; p-value):
- adjusted for season, age at baseline and follow-up

Socio-demographic and biological correlates
(3) Dutch parental ethnicity (β=21.28; CI: 0.56, 42.00; $p<0.05$) is positively associated with outdoor play in children at baseline (5 yrs); no association at 2 yrs follow-up ($p>0.05$)

Parents' psychological, cognitive and emotional correlates
(5) negative association of parental attitude to improve child engagement in outside play (agree) and outdoor play in children at baseline (5 yrs) (β=-5.38; CI: -9.32, -2.34; $p<0.05$) and at 2 yrs follow-up (-5.74; CI: -9.95, -1.52; $p<0.05$)

(7) negative association of parental intention to improve child engagement in outside play (agree) and outdoor play in children at baseline (5 yrs) (β=-11.33; CI: -18.01, -4.66; $p<0.05$); no association at 2 yrs follow-up ($p<0.05$)

Parenting practices
(9) positive association of presence of rules (β=29.71; CI: 23.89, 35.54; $p<0.05$) and outdoor play in children at baseline (5 yrs) and at 2 yrs follow-up (β=23.85; CI: 17.04, 30.66; $p<0.05$)

15	**Remmers, Van Kann et al. (2014) [69]**<br><br>Netherlands	**Study design**<br>longitudinal study<br><br>**Data collection**<br>Oct 2000 - 2005 (from prospective cohort study (KOALA Birth Cohort Study))<br><br>**Participants**<br>n = 1,875<br>5 yrs (5.0 yrs - baseline); 7 yrs (7.0 yrs- follow - up)<br>49% (F) (baseline)<br>rr = n.r.	**Definition**<br>PA without any given tasks or goals; unstructured free play; PA domain, that has been recommended to increase PA in children [115]<br><br>**Measurement parental report**:<br>*how many days the child played outside in an average week* ('never or less then one day on average' till 'seven days per week')<br><br>*indication of the average duration of outdoor play* ('shorter than half an hr'; 'half to one hr'; 'one to two hrs'; two to three hrs'; 'three hrs or more')	**Parents' psychological, cognitive and emotional correlates**<br>**(1)** parental attitude towards child's PA<br>**(2)** parental concerns (i.e. child's lack of PA, getting overweight)<br>**(3)** perceived responsibility for PA<br><br>**Parenting practices**<br>**(4)** parental TV restrictions<br>**(5)** pressure towards child to be active<br>**(6)** parental supervision of outdoor play<br><br>**Parents' social and cultural correlates**<br>**(7)** parent's social capital<br><br>**Parents' perceived neighborhood physical environment**<br>**(8)** perceived functionality<br>**(9)** perceived traffic safety<br>**(10)** perceived overall attractiveness of physical environment<br>**(11)** accessibility of number of facilities for PA<br><br>**Measurements parental report:**<br>**(1) - (6)** Child Feeding Questionnaire (CFQ) (Cronbach's α range= 0.57-0.93) [116]<br><br>**(7)** empirical researches (Cronbach's α=0.87) [117]	**Multivariate linear regression** (β coefficient; 95% CI; p-value):<br>- adjusted for gender, maternal education, child's age, functionality, traffic safety, attractiveness, accessibility, attitude, perceived responsibility, concern, restriction, pressure, monitoring, social capital<br><br>**Parents' psychological, cognitive and emotional correlates**<br>**(1)** positive association of parental attitude towards child PA (β=0.09; CI: 0.05, 0.13; p<0.01) and outdoor play in children<br><br>**(2)** negative association of parental concerns (β=-0.04; CI: -0.09, -0.001; p<0.05) and outdoor play in children<br><br>**(3)** no association of perceived responsibility for PA and outdoor play in children (p>0.05)<br><br>**Parenting practices**<br>**(4)** negative association of restrictions (β=-0.21; CI: -0.26, -0.17; p<0.01) and outdoor play in children<br><br>**(5)** and **(6)** no association pressure towards child to be active; supervision and outdoor play in children (p>0.05)<br><br>**Parents' social and cultural correlates**	*high*

				**(8) - (11)** Neighborhood Environment Walkability Scale (Cronbach's α range = 0.70-0.80; for component analyses 0.60) [118]	**(7)** positive association of parent's social capital (β=0.07; CI: 0.03, 0.11; p<0.01) and outdoor play in children<br><br>**<u>Parents' perceived neighborhood physical environment</u>**<br>**(8) - (10)** no significant association of parental perceived functionality; traffic safety; attractiveness and outdoor play in children (p>0.05)<br><br>**(11)** positive association of parent perceived accessibility of number of facilities for PA (β=0.05; CI: 0.01, 0.09; p<0.05) and outdoor play in children	
**16**	**Tandon et al. (2012) [55]**<br><br>United States	**<u>Study design</u>**<br>cross-sectional<br><br>**<u>Data collection</u>**<br>2001 - 2008 (from longitudinal, observational study - Early Childhood Longitudinal Study – Birth Cohort (ECLS-B))<br><br>**<u>Participants</u>**<br>n = 8,950	**<u>Definition</u>**<br>n.r.<br><br>**<u>Measurement</u>**<br>**parental report:**<br>*'In the past month, how often did you take [child] outside for a walk or to play in yard, a park, or a playground?'* ('once a day or more'; 'few times a week'; 'few times a month'; 'rarely' or 'not at all')	**<u>Socio-demographic and biological correlates</u>**<br>**(1)** maternal ethnicity<br>**(2)** maternal work status<br>**(3)** parental education<br><br>**<u>Parents' social and cultural correlates</u>**<br>**(4)** maternal PA<br><br>**<u>Measurements</u>**<br>**parental report**	**Multivariate models** (OR; 95% CI):<br>- adjusted for child's sex, number of regular playmates, screen time, type of primary child care arrangement, mother's race/ethnicity, mother's employment status/hours per week worked, parent's reported exercise frequency, highest attainment in the household<br><br>**<u>Socio-demographic and biological correlates</u>**<br>**(1)** Asian mothers had 49% (OR=0.51; CI: 0.43, 0.61) lower odds; black mothers had 41% (OR=0.59; CI: 0.49, 0.70) lower odds; Hispanic mothers had 20% (OR=0.80; CI: 0.67, 0.95) lower odds of taking their child outside daily (ref white mothers); no association for other ethnicities	*fair*

		preschool aged children (4.37 yrs)<br>49% (F)<br>rr = n.r.			**(2)** mothers who worked part time (<35h) had 18% (OR=0.82; CI: 0.70, 0.96) lower odds; full time (≥35h) had 30% (OR=0.70,CI: 0.62, 0.81) lower odds of taking their child outside daily (ref mothers who did not work outside the home)<br><br>**(3)** families for whom the highest educational attainment was more than high school (some college: OR=0.79; CI: 0.68, 0.91/ college graduate: OR=0.81; CI: 0.67, 0.99/ graduate degree: OR=0.84; CI: 0.71, 0.99) had lower odds of taking their child outside daily<br><br>**<u>Parents' social and cultural correlates</u>**<br>**(4)** mothers who exercised 4-7 times per week had 50% (OR=1.50; CI: 1.28, 1.75); 1-3 times per week had 5% (OR=1.05; CI: 0.89, 1.24) greater odds of taking their child outside daily (ref mothers who did not exercise); no association for exercise 1-3 times/week	
**17**	**Wijtzes et al. (2014) [66]**<br><br>Netherlands	**<u>Study design</u>**<br>cross-sectional<br><br>**<u>Data collection</u>**<br>Apr 2002 - Jan 2006<br><br>**<u>Participants</u>**<br>n = 4,726<br>6 yrs (6.08 yrs)<br>49.5% (F)<br>*rr = 85.2%*	**<u>Definition</u>**<br>n.r.<br><br>**<u>Measurement</u>**<br>**parental report:**<br>'*On average, how many weekdays/ weekend days per week does your child play outside*?'<br>(Likert-scale: 'never on weekdays/	**<u>Socio-demographic and biological correlates</u>**<br>**(1)** maternal education level<br>**(2)** paternal education level<br>**(3)** maternal employment status<br>**(4)** paternal employment status<br>**(5)** household income<br>**(6)** maternal ethnicity<br><br>**<u>Measurements</u>**<br>**parental report:**	**Multivariate models** (OR; 95% CI; p-value):<br>- adjusted for ethnicity, child's age, season, SEP indicators<br><br>**<u>Socio-demographic and biological correlates</u>**<br>**(1)** negative association of low maternal education (OR=0.67; CI: 0.49, 0.92; p<0.05) and outdoor play <1 hr/day in children (ref high education)<br><br>**(2)** no significant association of paternal education and outdoor play in children (p>0.05)	***high***

			weekend days' to 'every weekday/ weekend day'); '*On the days that your child plays outside, how long, on ave-rage, does your child play outside?* differentiation between week- and weekend days ('Never' to '3-4 hrs')	**(1) - (2)** Dutch Standard Classification of Education [119]<br><br>**(3) - (4)** response options: 'no paid job'/'paid job'	**(3) - (4)** no significant association of paternal or maternal employment status and outdoor play in children (p>0.05)<br><br>**(5)** positive association of <€ 2000/mo. income and outdoor play <1 hr/day in children (OR=1.57; CI: 1.21, 2.04; p<0.05) (ref 3,200€/mo)<br><br>**(6)** positive association of outdoor play (<1 hr/ day) in ethnic minority children compared with native Dutch children (expect Surinamese-Creole) (OR-range=1.73-3.55; CI-range: 1.08, 4.69; p<0.05)	
**18**	**Wilkie et al. (2018) [57]**<br><br>United Kingdom	<u>**Study design**</u><br>cross-sectional<br><br><u>**Data collection**</u><br>Sep 2011 - Jan 2013 (from International Study of Childhood Obesity, Lifestyle and the Environment (ISCOLE))<br><br><u>**Participants**</u><br>n = 462<br>9-11 yrs (10.9 yrs)	<u>**Definition**</u><br>n.r.<br><br><u>**Measurement**</u><br>**child's report:**<br>*time spend outside 'on a school day after school before bedtime' and 'on a weekend day'* ('<1hr'; '1hr'; '2hrs'; '3hrs'; '4hrs'; '5 or more hrs' (Diet and Lifestyle Questionnaire [120]	<u>**Socio-demographic and biological correlates**</u><br>**(1)** parental education (indicator of socioeconomic status - SES)<br><br><u>**Parents' perceived neighborhood physical environment**</u><br>**(2)** traffic related safety concerns<br><br><u>**Parents' perceived neighborhood social environment**</u><br>**(3)** crime-related safety concerns<br><br><u>**Measurements**</u><br>**parental report:**<br>**(2)** and **(3)** crime-related and traffic related safety concerns (10 items): Neighborhood Environment Walkability Scale for Youth [118]	**Multivariate logistic regression** (OR; 95% CI; p-value):<br>- adjusted for age, sex, SES<br><br><u>**Socio-demographic and biological correlates**</u><br>**(1)** positive association of low SES (OR=1.77; CI: 1.12, 2.80; p=0.015) and outdoor play in children (Ref. high SES) for weekdays; no association for weekend days (p>0.05)<br><br><u>**Parents' perceived neighborhood physical environment**</u><br>**(2)** no association of traffic-related parental safety concerns and outdoor play in children for week- and weekend days (p>0.05)<br><br><u>**Parents' perceived neighborhood social environment**</u>	***high***

		girls: 55%<br>rr = n.r.			**(3)** positive association of crime-related parental safety concerns (OR=1.51; CI: 1.09, 2.10; p=0.013) and outdoor play in children on weekdays; no association of crime related parental safety concerns and outdoor play in children for weekend days (p>0.05)	
**19**	**Xu et al. (2016) [70]**<br><br>Australia	**Study design**<br>longitudinal study<br><br>**Data collection**<br>2007 - 2013 (from Healthy Beginning Trial (HBT))<br><br>**Participants**<br>n = 667<br>2-5 yrs (n.c.)<br>49-50% (F)<br>rr = n.r.	**Definition**<br>often used as a proxy of PA of young children [95]<br><br>**Measurement**<br>**maternal report:**<br>*how much time the child spent playing outdoors on a typical weekday and on a typical weekend day* [95]	**Socio-demographic and biological correlates**<br>**(1)** maternal ethnicity<br><br>**Psychological, cognitive and emotional correlates**<br>**(2)** mother's information about playing with children<br>**(3)** mother's knowledge of child development<br>**(4)** mother's awareness of childhood obesity<br>**(5)** belief of being able to give child activities<br><br>**Parenting practices**<br>**(6)** rules for TV watching<br><br>**Parents' social and cultural correlates**<br>**(7)** maternal PA<br><br>**Measurements**<br>**parental report:**<br><br>**(3)** assessed by 3 questions (i.e. 'How informed do you feel about child development e.g. age child typically	**Multivariate models** (β coefficient; 95% CI / OR; CI):<br>- adjusted for time, time², intervention allocation, child's sex, mothers' country of birth, mother's education status at baseline, child-care attendance, mothers' employment status, TV time rules, interaction of TV time rules and time at 2, 3.5 and 5 yrs<br><br>**Socio-demographic and biological correlates**<br>**(1)** children whose mothers were Australian born (CI: 14.3, 36.2) had 25 min greater playtime outdoors per day (Ref. Other)<br><br>**Psychological, cognitive and emotional correlates**<br>**(2)** being informed about playing with children is positively associated with daily (β=11.6; CI: 1.56, 21.54); on weekend days (β=14.9; CI: 1.75, 28.05) outdoor play in children across ages 2, 3.5 and 5 yrs; no association for weekdays<br><br>**(3) - (5)** no significant association of mother's knowledge of child development; being able to give child activities; mother's awareness of childhood obesity and outdoor play in children	***fair***

				crawls, walk, runs?'; 'How able do you feel you are to give your child activities that will keep them occupied while you are doing other things?')<br><br>**(4)** assessed by one question ('How worried should adults be about their children being overweight or obese?')<br><br>**(6)** NSW Child Health Survey 2001 [121]<br><br>**(6)** assessed by 3 questions (i.e. 'Are there rules about what TV programs your child can watch?'; 'Are there rules about how many hours of TV your child can watch?' [122]<br><br>**(7)** Questions from the Active Australia Survey questionnaire 2003 [123]	**<u>Parenting practices</u>**<br>**(6)** children from families carrying TV time rules had 22 min (CI: 8.8, 35.5) more outdoor playtime per day at age 2 yrs; no association at ages 3.5 and 5 yrs<br><br>**<u>Social and cultural correlates</u>**<br>**(7)** each one hour increase in mother's physical activity time before pregnancy is associated with 6 min (95 % CI 2.06–11.04) more children's outdoor playtime on a weekend day; no significant association of maternal PA (h/day) and daily (β=3.9; CI: 0.46, 7.28); weekday (β=2.7; CI: -0.70, 6.18) outdoor play in children across ages 2, 3.5 and 5 yrs<br><br>**(7)** no significant association of maternal baseline PA (≥150min/week) and daily (β=0.37; CI: -10.53, 11.28); weekday (β=-0.8; CI: -11.78, 10.13); on weekend days (β=5.3; CI: -9.15, 19.67) outdoor play in children across ages 2, 3.5 and 5 yrs	
**20**	**Xu et al. (2017) [26]**<br><br>Australia	**<u>Study design</u>**<br>cross-sectional<br><br>**<u>Data collection</u>**<br>2007 - 2013<br>(from Healthy Beginning Trial (HBT))	**<u>Definition</u>**<br>primary form of PA for young children [60]; usually free play without goals set by adults, but under parental or	**<u>Socio-demographic and biological correlates</u>**<br>**(1)** maternal education level<br>**(2)** maternal ethnicity<br><br>**<u>Parents' social and cultural correlates</u>**<br>**(3)** maternal PA	**Multivariate logistic regression** (OR; 95% CI):<br>- adjusted for intervention allocation, child's sex, mother's country of birth, education level, PA level, childcare attendance at 2 and 3.5 yrs<br><br>**<u>Socio-demographic and biological correlates</u>**	*fair*

	 <u>Participants</u><br>n = 497<br>2-3.5 yrs (n.c.)<br>49-50% (F)<br>rr = n.r.	carer's supervision [124]<br><br>**<u>Measurement</u>**<br>**maternal report**:<br>*how much time the child spent playing outdoors on a typical weekday and on a typical weekend day* [95]	**<u>Parents' perceived neighborhood physical environment</u>**<br>**(4)** perceptions of good parks or playgrounds in the neighborhood<br>**(5)** perceptions of heavy traffic<br>**(6)** perceptions of neighborhood as a good place to bring up children<br><br>**<u>Parents' perceived neighborhood social environment</u>**<br>**(7)** perceptions of child's safety during outdoor play<br>**(8)** perceived neighborhood safety<br><br>**<u>Measurements</u>**<br>**maternal report:**<br>**(4) - (8)** Growing up in Australia: The Longitudinal Study of Australian Children [125]	**(1)** children with mothers with higher education were less likely to play outdoors $\geq$ 2 h/day on weekdays (no values are mentioned in study)<br><br>**(2)** children with Australian born mothers were more likely to play outdoors $\geq$2 h/day on weekdays and weekend days (2-3.5 yrs; no values are mentioned in study)<br><br>**<u>Parents' social and cultural correlates</u>**<br>**(3)** no significant association of maternal PA and outdoor play in children on weekdays and weekend days (at age 2 or 3.5) ($p>0.05$)<br><br>**<u>Parents' perceived neighborhood physical environment</u>**<br>**(4)** maternal perception of good parks or playgrounds in the neighborhood is positively associated with playing outdoors for $\geq$2 h/day on weekdays (OR=1.86; CI: 1.09, 3.18), and on weekends (OR=1.83; CI: 1.03, 3.25) at age 2; no association at age 3.5 ($p>0.05$)<br><br>**(5)** no association of traffic situation in neighborhood and playing outdoors for $\geq$2 h/day on weekdays and weekend days (at age 2 and 3.5 yrs) ($p>0.05$)<br><br>**(6)** maternal perception of neighborhood as a good place to bring up children is positively associated with playing outdoor for $\geq$2 h/day on weekdays (OR=1.87;CI: 1.13, 3.07) and on weekends (OR=1.91; CI: 1.12, 3.27) at age 2 and

					on weekend days (OR 2.96; CI: 1.42, 6.17) at age 3.5; no association for weekdays (3.5 yrs)<br><br>**Parents' perceived neighborhood social environment**<br>**(7)** maternal perception of child's safety during outdoor play is positively associated with playing outdoor for≥2 h/day on weekdays (OR=2.06; CI: 1.29, 3.30), and on weekends (OR=2.47, CI: 1.46, 4.19) at age 2 and on weekends (OR= 1.94; CI: 1.02, 3.70) at age 3.5; no association for weekdays (3.5 yrs)<br><br>**(8)** no association of maternal perceived neighborhood safety and playing outdoors for ≥2 h/day on weekdays and weekend days (at age 2 and 3.5 yrs) (p>0.05)	
**21**	**Xu et al. (2014) [67]**<br><br>Australia	**Study design**<br>cross-sectional<br><br>**Data collection**<br>2007 - 2010 (from Healthy Beginning Trial (HBT))<br><br>**Participants**<br>n = 497<br>2 yrs (n.c.)<br>50 % (F)<br>rr = 75%	**Definition**<br>principal form of PA; used as a proxy in measuring PA of young children [95]<br><br>**Measurement**<br>**maternal report:**<br>*'How much time would you say your child spends playing outdoors on a typical week-/ weekend day?'* (separately for week- and weekend days) [95]	**Socio-demographic and biological correlates**<br>**(1)** maternal age<br>**(2)** maternal marital status<br><br>**Parents' psychological, cognitive and emotional correlates**<br>**(3)** parental hostility<br><br>**Parents' perceived neighborhood social environment**<br>**(4)** perception of safety during outdoor play<br><br>**Measurements**<br>**maternal report:**	**Multivariate logistic regression** (OR; 95% CI; p-value):<br>- adjusted for effect of intervention, baby gender, mothers' age, marital status, parental hostility, safe outdoor play<br><br>**Socio-demographic and biological correlates**<br>**(1)** mother's age: 25-29 yrs (OR=0.62; CI: 0.38, 1.02; p= 0.028); ≥30 yrs (OR=0.50; CI: 0.30, 0.84; p=0.028) is negatively associated with ≥2 h/day playtime outdoors (Ref. 16-24 yrs)<br><br>**(2)** mothers' marital status (married) (OR=0.35; CI: 0.14, 0.85; p=0.021) is negatively associated with ≥2 h/day playtime outdoors (Ref. other)	*high*

	**(3)** and **(4)** Growing up in Australia: The Longitudinal Study of Australian Children [126]	**Parents' psychological, cognitive and emotional correlates**<br>**(3)** positive association of low parental hostility (OR=2.65; CI: 1.68, 4.02; p<0.0001) and ≥2 h/day playtime outdoors (Ref. high)<br><br>**Parents' perceived neighborhood social environment**<br>**(4)** positive association of perception that outdoor play is safe (OR=2.44; CI: 1.55, 3.85; p<0.0001) and ≥2 h/day playtime outdoors (ref no)

Note: PA= physical activity; MVPA = moderate-to-vigorous physical activity; BMI= body mass index; rr = response rate; yrs = years; mo. (s) = month/months; n.r. = not reported; n.c. = not calculable; hr(s) = hour(s); min = minutes; italic and in green colored indications = self calculated
