# Supplementary material for: Parental Correlates of Outdoor Play in Boys and Girls Aged 0 to 12—A Systematic Review
Source: Int J Environ Res Public Health. 2019 Jan 11;16(2):190. doi: 10.3390/ijerph16020190 (PMC6351982; doi:10.3390/ijerph16020190)
Supplement: Supplementary file 1 [file ijerph-16-00190-s001.zip › ijerph-397145 Supplementary - For Proof/ijerph-397145 Table S2.pdf]

Table S2. Extracted data of studies included in systematic review (gender-related findings).

#	**Author (year)**<br><br>**Country**	**Study design / Data collection / Participants** (sample size; age range (mean); gender; response rate)	**Outdoor Play**<br><br>**Definition**<br><br>**Measurement**	**Parental correlates**<br><br>**Measurement**	**Main findings of gender differences**	**Study quality**
1	**Aarts et al. (2010) [56]**<br><br>Netherlands	**Study design**<br>cross-sectional<br><br>**Data collection**<br>Sep 2007 - Jan 2008<br><br>**Participants**<br>n = 6,470<br>4-12 yrs (*7.9 yrs*)<br>*49.9% (F)*<br>rr = 60%	**Definition**<br>natural way for children to be physically active [56]; in contrast to organized sports, outdoor play is cheap, informal and easily accessible [8]<br><br>**Measurement**<br>**parental report:** (considering a typical week in the last mo./separately questions for week- and week-end days): *'How many days does your child play outside on week-days/weekends?'*; '*On average, how long does your child*	**Parents' perceived neighborhood physical environment**<br>**(1)** degree of unoccupied houses<br>**(2)** presence of dog waste<br>**(3)** presence of water<br>**(4)** diversity of routes<br><br>**Parents' perceived neighborhood social environment**<br>**(5)** social cohesion<br><br>**Measurements**<br>**parental report:** response options: Five-point Likert scale from 'strongly agree' to 'strongly disagree' [93]	**Multivariate GEE analysis** (Relative rate; 95% CI): - adjusted for parental education<br><br>**Parents' perceived neighborhood physical environment**<br>**(1)** positive association of the degree of unoccupied houses and outdoor play in boys (10-12 yrs, RR = 1.05, CI: 1.02, 1.08); no association for girls (10-12 yrs)<br><br>**(2)** positive association of the presence of dog waste and outdoor play in girls (4-6 yrs, RR = 1.03, CI: 1.00, 1.05); no association for boys (4-6 yrs)<br><br>**(3)** positive association of the presence of water and outdoor play in boys (4-6 yrs, RR = 1.04, CI: 1.01, 1.07); no association for girls (4-6 yrs)<br><br>**(4)** positive association of the perceived diversity of routes and outdoor play in girls (7-9 yrs, RR = 1.03, CI: 0.99, 1.06) and boys (10-12 yrs, RR = 1.08, CI: 1.03, 1.13); no association for boys (7-9 yrs) and girls (10-12 yrs)	*high*

			spend on out-door play on such a weekday/weekend day?' [93]		**<u>Parents' perceived neighborhood social environment</u>**<br>**(5)** positive association of perceived social cohesion and outdoor play in girls (10-12 yrs, RR = 1.02, CI: 1.01, 1.04); no association for boys (10-12 yrs)	
**2**	**Cleland et al. (2010) [23]**<br><br>Australia	**<u>Study design</u>**<br>longitudinal<br><br>**<u>Data collection</u>**<br>Jul - Dec 2001, 2004 and 2006 (The Children Living in Active Neighborhoods (CLAN) study)<br><br>**<u>Participants</u>**<br>n = 421<br>5-6 and 10-12 yrs at baseline (n.c.)<br>n.c. (F)<br>rr = 38.3%	**<u>Definition</u>**<br>n.r.<br><br>**<u>Measurement</u>**<br>**parental report:**<br>*'In total, how many hrs/min does your child usually spend outside during a typical week after school?'* (separately for week- and weekend days)	**<u>Parenting practices</u>**<br>**(1)** outdoor social opportunities<br>**<u>Parents' social and cultural correlates</u>**<br>**(2)** parental encouragement<br><br>**<u>Measurements</u>**<br>**parental report:**<br>**(1)** four statements about who the child goes outside with (ICC 0.61-0.79)<br><br>**(2)** responses to two statements about encouragement of and success in getting the child to play outside (ICC 0.42-0.54)	**Longitudinal linear regression** (adjusted β coefficient; 95% CI; p-value):<br>- adjusted for maternal education, parental marital status, outdoor tendencies, parental encouragement, social opportunities, supervision, dog ownership, yard size, home PA opportunities, weather<br><br>**<u>Parenting practices</u>**<br>**(1)** high social opportunities (β=169.7; CI: 25.9, 313.6; p<0.05) are positively associated with average weekly time (minutes/week) spent outdoors in boys (5-6 yrs) (ref low); no association of high social opportunities and outdoor play (p>0.05) for girls (5-6 yrs)<br><br>**<u>Social and cultural correlates</u>**<br>**(2)** high parental encouragement (β=234.0; CI: 30.1, 437.8; p<0.05) is positively associated with average weekly time (minutes/week) spent outdoors in girls (5-6 yrs) (ref low); no association of high parental encouragement and outdoor play (p>0.05) for boys (5-6 yrs)<br><br>**(2)** high parental encouragement (β=151.4; CI: 66.6, 236.2; p<0.01) is positively associated with	*fair*

					average weekly time (minutes/week) spent outdoors in girls (10-12 yrs) (ref low); no association of high parental encouragement and outdoor play (p>0.05) for boys (10-12 yrs)	
3	**Nicksic et al. (2018) [48]**<br><br>United States	**<u>Study design</u>**<br>cross-sectional<br><br>**<u>Data collection</u>**<br>2012 - 2014 (from Texas Childhood Obesity Research Demonstration (TX CORD))<br><br>**<u>Participants</u>**<br>n = 748<br>grade 5 (n.r.)<br>57.9% (F)<br>rr = ~90%	**<u>Definition</u>**<br>n.r.<br><br>**<u>Measurement</u>**<br>**parental report:** *'Most of the time does your child play outdoors for at least 30 min per day? Do not count outdoor play during school hrs.'*<br><br>**child's report:** *'Last week, on what days did you play out-doors for at least 30 min or more? Do not count outdoor play during school hrs.'*(Spearman's correlation bet-ween child's and parents report 0.168; p=0.01)	**<u>Parents' social and cultural correlates</u>**<br>**(1)** parental encouragement<br><br>**<u>Parents' perceived neighborhood social environment</u>**<br>**(2)** neighborhood safety<br><br>**<u>Measurements</u>**<br>**parental report:**<br>**(1)** extent of agreement with the statement 'If the weather is good, I encourage my child to play outside.'; (Likert scale: 'strongly disagree' to 'strongly agree') [109]<br><br>**(2)** 11-item scale (Cronbach's α= 0.91); adapted from the Teen Health 2000 Study [110]	**Multivariate models** (β coefficient; p-value):<br>- adjusted for sociodemographic variables, child's weight status, parent's PA<br><br>**<u>Social and cultural correlates</u>**<br>**(1)** positive association of parental encouragement and child's reported outdoor play in girls (β=0.650; p=0.018); no significant association for boys (p>0.05)<br><br>**<u>Parents' perceived neighborhood social environment</u>**<br>**(2)** positive association of perceived neighborhood safety (β=0.667; p=0.033) and child's reported outdoor play in boys; no association for girls (p>0.05)	***high***
4	**Wilkie et al. (2018) [57]**	**<u>Study design</u>**<br>cross-sectional<br><br>**<u>Data collection</u>**	**<u>Definition</u>**<br>n.r.<br><br>**<u>Measurement</u>**	**<u>Parents' perceived neighborhood physical environment</u>**<br>**(1)** traffic related safety concerns	**Multivariate logistic regression** (OR; 95% CI; p-value):<br>- adjusted for age, sex, SES	***high***

United Kingdom	Sep 2011 - Jan 2013 (from International Study of Childhood Obesity, Lifestyle and the Environment (ISCOLE))<br><br>**<u>Participants</u>**<br>n = 462<br>9-11 yrs (10.9 yrs)<br>*girls: 55%*<br>rr = n.r.	**child's report:**<br>*time spend outside 'on a school day after school before bedtime' and 'on a weekend day'* ('<1hr'; '1hr'; '2hrs'; '3hrs'; '4hrs'; '5 or more hrs' (Diet and Lifestyle Questionnaire [120]	**<u>Measurements</u>**<br>**parental report:**<br>**(1)** traffic related safety concerns (10 items): Neighborhood Environment Walkability Scale for Youth [118]	**<u>Parents' perceived neighborhood physical environment</u>**<br>**(1)** per increased unit in the traffic-related safety concerns score lower odds of spending more time outdoors after school in boys only (OR=0.52;CI: 0.28, 0.97; p=0.040) were found; no significant association for girls (OR=1.36, CI: 0.84, 2.21; p=0.207)

Note: PA= physical activity; MVPA = moderate-to-vigorous physical activity; BMI= body mass index; rr = response rate; yrs = years; mo. (s) = month/months; n.r. = not reported; n.c. = not calculable; hr(s) = hour(s); min = minutes; italic and in green colored indications = self calculated
