# Supplementary material for: Parental Correlates of Outdoor Play in Boys and Girls Aged 0 to 12—A Systematic Review
Source: Int J Environ Res Public Health. 2019 Jan 11;16(2):190. doi: 10.3390/ijerph16020190 (PMC6351982; doi:10.3390/ijerph16020190)
Supplement: Supplementary file 1 [file ijerph-16-00190-s001.zip › ijerph-397145 Supplementary - For Proof/ijerph-397145 Table S3.pdf]

Table S3. Quality assessment for all included studies [a, b]

		Introduction	Methods										Results					Discussion		Study quality	
#	Author (year)	1. Objectives	2. Study design	3. Sample size justification	4. Definition of the target population	5. Sampling frame	6. Sample selection process	7. Address of non-responders	8. Appropriateness aim(s) and outcome	9. Appropriateness of measurements	10. Clearness of statistical significance	11. Sufficiently description of methods	12. Adequately description of basic data	13. Response rate	14. Description of non-responders	15. Consistent results	16. Completeness of results	17. Justified results	18. Limitations	Study quality score (percentage)	Study quality rating
1	**Aarts et al. (2012) [58]**	1	1	0	1	1	0.5	0	1	0	*0.75*	*0.75*	1	1	*0.75*	1	0.5	*0.75*	1	**13/18 (72.2)**	*high*
2	**Aarts et al. (2010) [56]**	1	1	1	1	1	0.5	0	1	0	*0.75*	*0.75*	1	1	0	1	0.5	0.5	1	**13/18 (72.2)**	*high*
3	**Bringolf-Isler et al. (2010) [59]**	1	*0.75*	0	1	0.5	0.5	0	1	0	1	*0.75*	0.5	1	0	*0.75*	*0.75*	1	0.5	**11/18 (61.1)**	*fair*
4	**Burdette et al. (2005) [60]**	0.5	1	0	1	*0.75*	0.5	0	*0.75*	0	*0.75*	1	1	0	0	1	1	1	1	**11.25/18 (62.5)**	*fair*
5	**Carsley et al. (2016) [61]**	1	*0.75*	0	1	0.5	1	0	1	0	*0.75*	1	1	0	0	1	1	1	1	**11.5/18 (63.9)**	*fair*
6	**Cleland et al. (2010) [23]**	*0.75*	1	0	1	0.5	1	0	1	0.5	0.5	1	0.5	0	0	1	0.5	1	1	**11.25/18 (62.5)**	*fair*
7	**Faulkner et al. (2015) [5]**	1	1	0	1	0.5	1	0	1	0	0.5	*0.75*	*0.75*	1	0	1	0.5	1	1	**11.5/18 (63.9)**	*fair*

#	Author (year)	1. Objectives	2. Study design	3. Sample size justification	4. Definition of the target population	5. Sampling frame	6. Sample selection process	7. Address of non-responders	8. Appropriateness aim(s) and outcome	9. Appropriateness of measurements	10. Clearness of statistical significance	11. Sufficiently description of methods	12. Adequately description of basic data	13. Response rate	14. Description of non-responders	15. Consistent results	16. Completeness of results	17. Justified results	18. Limitations	Study quality score (percentage)	Study quality rating
8	**Ferrao et al. (2015) [62]**	1	*0.75*	1	1	*0.75*	0.5	0	1	0.5	0	*0.75*	1	0	0	*0.75*	0	1	1	**11/18 (61.1)**	*fair*
9	**Janssen (2015) [27]**	1	*0.75*	1	1	0.5	0.5	0	1	0.5	1	1	1	0	0.5	1	1	*0.75*	1	**13/18 (72.2)**	*high*
10	**Kimbro et al. (2011) [63]**	1	*0.75*	0	1	1	*0.75*	0	1	*0.75*	1	1	0.5	1	1	1	0.5	*0.75*	1	**14/18 (77.8)**	*high*
11	**Marino et al. (2012) [64]**	1	*0.75*	0.5	1	1	*0.75*	0	1	0	1	1	1	0	*0.75*	1	1	1	1	**13.75/18 (76.3)**	*high*
12	**McDonald et al. (2015) [65]**	1	*0.75*	0	1	0.5	0.5	0	1	*0.75*	1	1	1	0	0	1	1	1	1	**12/18 (66.7)**	*high*
13	**Nicksic et al. (2018) [48]**	1	*0.75*	0	1	*0.75*	*0.75*	0	1	1	1	1	1	1	1	1	0.5	1	1	**14.75/18 (81.9)**	*high*
14	**Remmers, Broeren et al. (2014) [68]**	1	1	0	1	0.5	*0.75*	0	*0.75*	0.5	0.5	*0.75*	1	0	0.5	1	*0.75*	1	*0.75*	**11.5/18 (63.9)**	*fair*

#	Author (year)	1. Objectives	2. Study design	3. Sample size justification	4. Definition of the target population	5. Sampling frame	6. Sample selection process	7. Address of non-responders	8. Appropriateness aim(s) and outcome	9. Appropriateness of measurements	10. Clearness of statistical significance	11. Sufficiently description of methods	12. Adequately description of basic data	13. Response rate	14. Description of non-responders	15. Consistent results	16. Completeness of results	17. Justified results	18. Limitations	Study quality score (percentage)	Study quality rating
15	**Remmers, Van Kann et al. (2014) [69]**	1	1	0	1	*0.75*	0	0	1	0.5	1	1	1	0	1	1	0.5	1	1	**12.75/18 (70.8)**	*high*
16	**Tandon et al. (2012) [55]**	1	1	0	1	1	1	0	0.5	0	1	*0.75*	1	0	0	1	*0.75*	1	1	**11.25/18 (62.5)**	*fair*
17	**Wijtzes et al. (2014) [66]**	1	*0.75*	0.5	1	1	0.5	0	1	0	1	1	1	1	1	1	*0.75*	0.75	1	**14.25/18 (79.2)**	*high*
18	**Wilkie et al. (2018) [57]**	1	*0.75*	0	1	0.5	0.5	0	1	0	1	*0.75*	1	0	1	1	1	*0.75*	1	**12.25/18 (68.1)**	*high*
19	**Xu et al. (2016) [70]**	*0.75*	1	0	1	0.5	0.5	0	1	0.5	1	1	1	0	0.5	1	0	1	1	**11.75/18 (65.3)**	*fair*
20	**Xu et al. (2017) [26]**	1	*0.75*	0	1	0.5	0.5	0	1	0	1	1	1	0	0.5	1	0	1	1	**11.25/18 (62.5)**	*fair*
21	**Xu et al. (2014) [67]**	1	1	0	1	1	*0.75*	0	1	0	1	1	1	1	0	1	1	1	1	**13.75/18 (76.4)**	*high*

Note: italic written values are means of the quality assessment of both reviewers (BK, TM); a = rating points for quality assessment: criterion fulfilled = 1; partial fulfilled = 0.5; not fulfilled = 0; unclear = 0; not applicable = 0 (based on Bates & Stone [43]; Schoeppe et al. [42]); b = study quality rating: *>66.7%* (high study quality); *50-66.6%* (fair study quality); *<50%* (low study quality) (based on Newell, Bowman and Cockburn, [44]).
